# Supplementary material for: Korean Medicine Clinical Practice Guideline Update for Temporomandibular Disorders: An Evidence-Based Approach
Source: Healthcare (Basel). 2023 Aug 21;11(16):2364. doi: 10.3390/healthcare11162364 (PMC10454242; doi:10.3390/healthcare11162364)

**Supplementary legends**

Eq. (A.1): Composition of development committee (2p)

Eq. (B.1) ~ (B.9): Search strategy for the Patients/Populations and Intervention components (3~55p)

Eq. (C.1) ~ (C.8): PRISMA flow chart (55~62p)

Eq. (D.1) ~ (D.5): Forest plot & risk of bias table (63~74p)

Eq. (E.1) ~ (E.17): Evidence table (see ‘Supplementary Materials S2’ file)

**Eq. (A.1) Composition of development committee**

| Group | Member of name | Information of members | Affiliation |
| --- | --- | --- | --- |
| Working group | Jae-Heung Cho | Professor | Korean medicine Hospital, Kyung Hee University |
|  | In-Hyuk Ha | Researcher | Jaseng Medical Foundation |
|  | Yoon Jae Lee | Researcher | Jaseng Medical Foundation |
|  | Byung-cheul Shin | Professor | Pusan National University Korean Medicine Hospital |
|  | Eun-jung Kim | Professor | Korean Medicine Hospital, Dongguk University |
|  | Yeon-Cheol Park | Professor | Korean Medicine Hospital, Kyung Hee University |
|  | Koh-Woon Kim | Professor | Korean medicine Hospital, Kyung Hee University |
|  | Mi-Riong Kim | Researcher | Jaseng Korean medicine hospital |
|  | Jae-Young Jung | Clinical Practitioner | Mokhuri Korean medicine hospital |
| Review committee group | Seung-Ryong Yeom | Professor (Korean Academy of Oriental Rehabilitation Medicine) | Korean Medicine Hospital, Wonkwang University |
|  | Yun-Yeop Cha | Professor (Korean Academy of Oriental Rehabilitation Medicine) | Korean Medicine Hospital, Sangji University |
|  | Youn-Seok Ko | Professor (Korean Academy of Oriental Rehabilitation Medicine) | Korean Medicine Hospital, Woosuk University |
|  | Byung-Kwan Seo | Professor (Korea Acupuncture & Moxibustion Medicine Society) | Korean Medicine Hospital, Kyung Hee University |
|  | Hyun-Seok Cho | Professor (Korea Acupuncture & Moxibustion Medicine Society) | Korean Medicine Hospital, Dongguk University |
|  | Dongwoo Nam | Professor (Korea Acupuncture & Moxibustion Medicine Society) | Korean Medicine Hospital, Kyung Hee University |
|  | Won-Suk Sung | Professor (Korea Acupuncture & Moxibustion Medicine Society) | Korean Medicine Hospital, Dongguk University |
|  | Hyun-Young Kwak | Clinical Practitioner | Kyunghee DMC Clinic |
|  | Ha-Neul Kim | Clinical Practitioner | Jaseng Korean medicine hospital |
|  | You-Suk Youn | Clinical Practitioner | Mokhuri Korean medicine hospital |
|  | Jiae Choi | EBM specialist | Korea Health Promotion Institute |
|  | Junhwan Lee | Researcher | Korea Institute Oriental Medicine |

**Search strategy for the Patients/Populations and Intervention components**

**Eq. (B.1) Acupuncture and laser acupuncture (Q1-4)**

* Ovid-Medline

1 exp Temporomandibular Joint/

2 exp Temporomandibular Joint Disorders/

3 (temporomandibular or temporo mandibular or temporo­ mandibular).mp.

4 (TMJ or TMD or CMD).mp.

5 (craniomandibular disorder$ or cranio mandibular or cranio­mandibular disease$).mp.

6 exp masticatory muscles/

7 Mandibular Condyle/

8 exp mandibular diseases/

9 exp Craniomandibular Disorders/

10 exp Temporomandibular Joint Dysfunction Syndrome/

11 Myofascial Pain Syndromes/

12 masticatory muscle$.mp.

13 masseter muscle$.mp.

14 pterygoid muscle$.mp.

15 temporal muscle$.mp.

16 (arthralgia and temporomandibular).mp.

17 (myofascial pain or mpd).mp.

18 mandibular condyle$.mp.

19 temporal bone$.mp.

20 mandibular disease$.mp.

21 mandibular neoplasm$.mp.

22 prognathism.mp.

23 retrognathism.mp.

24 masticatory muscle pain.mp.

25 internal derangement.mp.

26 myofacial pain.mp.

27 disc displacement.mp.

28 (pain dysfunction syndrome or pds).mp.

29 myogenous pain.mp.

30 arthrogenous.mp.

31 localized myalgia.mp.

32 crepitation.mp.

33 costen$.mp.

34 (tooth adj5 grind$).mp.

35 (teeth adj5 grind$).mp.

36 (jaw adj5 clench$).mp.

37 (ear adj5 (click$ or pop$)).mp.

38 (jaw adj5 lock$).mp.

39 exp Acupuncture/

40 exp Acupuncture Therapy/

41 acupuncture.mp.

42 exp acupuncture, ear/

43 exp electroacupuncture/

44 exp meridians/

45 exp acupuncture points/

46 acupressure.mp.

47 electroacupuncture.mp.

48 meridian$.mp.

49 needling.mp.

50 (trigger adj3 point$).mp.

51 acup$ point$.mp.

52 or/39­51

53 exp Jaw Diseases/

54 (or/1­38) or 53

55 52 and 54

56 randomized controlled trial.pt.

57 controlled clinical trial.pt.

58 randomized.ab.

59 placebo.ab.

60 drug therapy.fs.

61 randomly.ab.

62 trial.ab.

63 groups.ab.

64 or/56­63

65 exp animals/ not humans.sh.

66 64 not 65

67 55 and 66

* Ovid-EMBASE

1 exp temporomandibular joint/

2 exp temporomandibular joint disorder/

3 (temporomandibular or temporo mandibular or temporomandibular).mp.

4 (TMJ or TMD or CMD).mp.

5 (craniomandibular disorder$ or cranio mandibular or craniomandibular disease$).mp.

6 exp masticatory muscle/

7 exp mandible condyle/

8 exp jaw disease/

9 masticatory muscle$.mp.

10 masseter muscle$.mp.

11 pterygoid muscle$.mp.

12 temporal muscle$.mp.

13 temporal muscle$.mp.

14 (arthralgia and temporomandibular).mp.

15 (myofascial pain or mpd).mp.

16 mandibular condyle$.mp.

17 temporal bone$.mp.

18 mandibular disease$.mp.

19 mandibular neoplasm$.mp.

20 prognathism.mp.

21 retrognathism.mp.

22 masticatory muscle pain.mp.

23 internal derangement.mp.

24 myogenous pain.mp.

25 arthrogenous.mp.

26 localized myalgia.mp.

27 crepitation.mp.

28 costen$.mp.

29 (tooth adj5 grind$).mp.

30 (teeth adj5 grind$).mp.

31 (jaw adj5 clench$).mp.

32 (ear adj5 (click$ or pop$)).mp.

33 (jaw adj5 lock$).mp.

34 exp acupuncture/

35 exp acupuncture needle/

36 exp auricular acupuncture/

37 exp electroacupuncture/

38 acupuncture.mp.

39 acupressure.mp.

40 electroacupuncture.mp.

41 meridian$.mp.

42 needling.mp.

43 (trigger adj3 point$).mp.

44 acup$ point$.mp.

45 or/1-33

46 or/34-44

47 45 and 46

48 Clinical trial/

49 Randomized controlled trial/

50 Randomization/

51 Single blind procedure/

52 Double blind procedure/

53 Crossover procedure/

54 Placebo/

55 Randomi?ed controlled trial$.tw.

56 Rct.tw.

57 Random allocation.tw.

58 Randomly allocated.tw.

59 Allocated randomly.tw.

60 (allocated adj2 random).tw.

61 Single blind$.tw.

62 Double blind$.tw.

63 ((treble or triple) adj blind$).tw.

64 Placebo$.tw.

65 Prospective study/

66 or/48-65

67 47 and 66

* Cochrane Library

#1 MeSH descriptor: [Temporomandibular Joint] explode all trees

#2 MeSH descriptor: [Temporomandibular Joint Disorders] explode all trees

#3 MeSH descriptor: [Mandibular Condyle] explode all trees

#4 MeSH descriptor: [Masticatory Muscles] explode all trees

#5 MeSH descriptor: [Mandibular Diseases] explode all trees

#6 MeSH descriptor: [Craniomandibular Disorders] explode all trees

#7 MeSH descriptor: [Temporomandibular Joint Dysfunction Syndrome] explode all trees

#8 MeSH descriptor: [Jaw Diseases] explode all trees

#9 (temporomandibular or temporo mandibular or temporo­mandibular):ti,ab,kw

#10 (TMJ or TMD or CMD):ti,ab,kw

#11 (craniomandibular disorder$ or craniomandibular or cranio­mandibular disease$):ti,ab,kw (Word variations have been searched)

#12 masticatory muscle$:ti,ab,kw

#13 masseter muscle?:ti,ab,kw

#14 pterygoid muscle?:ti,ab,kw

#15 temporal muscle?:ti,ab,kw

#16 (arthralgia and temporomandibular):ti,ab,kw

#17 mandibular condyle$:ti,ab,kw

#18 temporal bone$:ti,ab,kw

#19 mandibular disease$:ti,ab,kw

#20 mandibular neoplasm$:ti,ab,kw

#21 prognathism:ti,ab,kw

#22 retrognathism:ti,ab,kw

#23 masticatory muscle pain:ti,ab,kw

#24 internal derangement:ti,ab,kw

#25 myofacial pain:ti,ab,kw

#26 (pain dysfunction syndrome or pds):ti,ab,kw

#27 myogenous pain:ti,ab,kw

#28 arthrogenous:ti,ab,kw

#29 localized myalgia:ti,ab,kw

#30 crepitation:ti,ab,kw

#31 costen$:ti,ab,kw

#32 (tooth near/5 grind$):ti,ab,kw

#33 (teeth near/5 grind$):ti,ab,kw

#34 (jaw near/5 clench$):ti,ab,kw

#35 (ear near/5 (click$ or pop$)):ti,ab,kw

#36 (jaw near/5 lock$):ti,ab,kw

#37 #1 or #2 or #3 or #4 or #5 or #6 or #7 or #8 or #9 or #10 or #11 or #12 or #13 or #14 or #15 or #16 or #17 or #18 or #19 or #20 or #21 or #22 or #23 or #24 or #25 or #26 or #27 or #28 or #29 or #30 or #31 or #32 or #33 or #34 or #35 or #36

#38 MeSH descriptor: [Acupuncture] explode all trees

#39 MeSH descriptor: [Acupuncture Therapy] explode all trees

#40 MeSH descriptor: [Acupuncture, Ear] explode all trees

#41 MeSH descriptor: [Electroacupuncture] explode all trees

#42 MeSH descriptor: [Meridians] explode all trees

#43 MeSH descriptor: [Acupuncture Points] explode all trees

#44 acupuncture:ti,ab,kw

#45 acupressure:ti,ab,kw

#46 electroacupuncture:ti,ab,kw

#47 meridian$:ti,ab,kw

#48 needling:ti,ab,kw

#49 (trigger near/3 point$):ti,ab,kw

#50 acup$point$:ti,ab,kw

#51 #38 or #39 or #40 or #41 or #42 or #43 or #44 or #45 or #46 or #47 or #48 or #49 or #50

#52 #37 and #51

* Ovid-AMED

1 exp Temporomandibular joint/

2 exp Temporomandibular joint disease/

3 exp Mandibular disease/

4 exp Jaw/

5 exp Temporomandibular joint syndrome/

6 temporomandibular.mp.

7 temporo mandibular.mp.

8 (TMJ or TMD or CMD).mp.

9 (craniomandibular disorder$ or cranio mandibular).mp.

10 masticatory muscle$.mp.

11 masseter muscle$.mp.

12 pterygoid muscle$.mp.

13 temporal muscle$.mp.

14 (arthralgia and temporomandibular).mp.

15 (myofascial pain or mpd).mp.

16 mandibular condyle$.mp.

17 temporal bone$.mp.

18 mandibular disease$.mp.

19 mandibular neoplasm$.mp.

20 prognathism.mp.

21 retrognathism.mp.

22 masticatory muscle pain.mp.

23 internal derangement.mp.

24 myofacial pain.mp.

25 (pain dysfunction syndrome or pds).mp.

26 myogenous pain.mp.

27 arthrogenous.mp.

28 localized myalgia.mp.

29 crepitation.mp.

30 costen$.mp.

31 (tooth adj5 grind$).mp.

32 (teeth adj5 grind$).mp.

33 (jaw adj5 clench$).mp.

34 (ear adj5 (click$ or pop$)).mp.

35 (jaw adj5 lock$).mp.

36 or/1-35

37 exp Acupuncture/

38 exp Acupuncture Therapy/

39 acupuncture.mp.

40 exp electroacupuncture/

41 exp meridians/

42 acupressure.mp.

43 electroacupuncture.mp.

44 meridian$.mp.

45 needling.mp.

46 (trigger adj3 point$).mp.

47 acup$ point$.mp.

48 exp Acupoints/

49 exp Ear acupuncture/

50 or/37-49

51 36 and 50

**Eq. (B.2) Pharmacopuncture (including bee venom therapy) (Q5)**

* Ovid-Medline

1 exp Temporomandibular Joint/

2 exp Temporomandibular Joint Disorders/

3 (temporomandibular or temporo mandibular or temporo­ mandibular).mp.

4 (TMJ or TMD or CMD).mp.

5 (craniomandibular disorder$ or cranio mandibular or cranio­mandibular disease$).mp.

6 exp masticatory muscles/

7 Mandibular Condyle/

8 exp mandibular diseases/

9 exp Craniomandibular Disorders/

10 exp Temporomandibular Joint Dysfunction Syndrome/

11 Myofascial Pain Syndromes/

12 masticatory muscle$.mp.

13 masseter muscle$.mp.

14 pterygoid muscle$.mp.

15 temporal muscle$.mp.

16 (arthralgia and temporomandibular).mp.

17 (myofascial pain or mpd).mp.

18 mandibular condyle$.mp.

19 temporal bone$.mp.

20 mandibular disease$.mp.

21 mandibular neoplasm$.mp.

22 prognathism.mp.

23 retrognathism.mp.

24 masticatory muscle pain.mp.

25 internal derangement.mp.

26 myofacial pain.mp.

27 disc displacement.mp.

28 (pain dysfunction syndrome or pds).mp.

29 myogenous pain.mp.

30 arthrogenous.mp.

31 localized myalgia.mp.

32 crepitation.mp.

33 costen$.mp.

34 (tooth adj5 grind$).mp.

35 (teeth adj5 grind$).mp.

36 (jaw adj5 clench$).mp.

37 (ear adj5 (click$ or pop$)).mp.

38 (jaw adj5 lock$).mp

39 exp Jaw Diseases/

40 (or/1­38) or 39

41 exp Bee Venoms/

42 pharmacopuncture.mp.

43 bee venom acupuncture.mp.

44 exp Acupuncture Points/

45 exp Injections/

46 acupoint$.mp.

47 bee venom$.mp.

48 aquapuncture.mp.

49 exp Trigger Points/

50 aqua acupuncture.mp.

51 herbal acupuncture.mp.

52 point injection.mp.

53 hydro acupuncture.mp.

54 injection$.mp.

55 45 or 54

56 44 or 46 or 49

57 55 and 56

58 41 or 42 or 43 or 47 or 48 or 50 or 51 or 52 or 53 or 57

59 40 and 58

* Ovid-EMBASE

1 exp temporomandibular joint/

2 exp temporomandibular joint disorder/

3 (temporomandibular or temporo mandibular or temporomandibular).mp.

4 (TMJ or TMD or CMD).mp.

5 (craniomandibular disorder$ or cranio mandibular or craniomandibular disease$).mp.

6 exp masticatory muscle/

7 exp mandible condyle/

8 exp jaw disease/

9 masticatory muscle$.mp.

10 masseter muscle$.mp.

11 pterygoid muscle$.mp.

12 temporal muscle$.mp.

13 temporal muscle$.mp.

14 (arthralgia and temporomandibular).mp.

15 (myofascial pain or mpd).mp.

16 mandibular condyle$.mp.

17 temporal bone$.mp.

18 mandibular disease$.mp.

19 mandibular neoplasm$.mp.

20 prognathism.mp.

21 retrognathism.mp.

22 masticatory muscle pain.mp.

23 internal derangement.mp.

24 myogenous pain.mp.

25 arthrogenous.mp.

26 localized myalgia.mp.

27 crepitation.mp.

28 costen$.mp.

29 (tooth adj5 grind$).mp.

30 (teeth adj5 grind$).mp.

31 (jaw adj5 clench$).mp.

32 (ear adj5 (click$ or pop$)).mp.

33 (jaw adj5 lock$).mp.

34 or/1-33

35 exp Bee Venoms/

36 pharmacopuncture.mp.

37 bee venom acupuncture.mp.

38 exp Acupuncture Points/

39 exp Injections/

40 acupoint$.mp.

41 bee venom$.mp.

42 aquapuncture.mp.

43 exp Trigger Points/

44 aqua acupuncture.mp.

45 herbal acupuncture.mp.

46 point injection.mp.

47 hydro acupuncture.mp.

48 injection$.mp.

49 39 or 48

50 38 or 40 or 43

51 49 AND 50

52 35 or 36 or 41 or 42 or 44 or 45 or 46 or 47 or 51

53 34 AND 52

* Cochrane Library

#1 MeSH descriptor: [Temporomandibular Joint] explode all trees

#2 MeSH descriptor: [Temporomandibular Joint Disorders] explode all trees

#3 MeSH descriptor: [Mandibular Condyle] explode all trees

#4 MeSH descriptor: [Masticatory Muscles] explode all trees

#5 MeSH descriptor: [Mandibular Diseases] explode all trees

#6 MeSH descriptor: [Craniomandibular Disorders] explode all trees

#7 MeSH descriptor: [Temporomandibular Joint Dysfunction Syndrome] explode all trees

#8 MeSH descriptor: [Jaw Diseases] explode all trees

#9 (temporomandibular or temporo mandibular or temporo­mandibular):ti,ab,kw

#10 (TMJ or TMD or CMD):ti,ab,kw

#11 (craniomandibular disorder$ or craniomandibular or cranio­mandibular disease$):ti,ab,kw (Word variations have been searched)

#12 masticatory muscle$:ti,ab,kw

#13 masseter muscle?:ti,ab,kw

#14 pterygoid muscle?:ti,ab,kw

#15 temporal muscle?:ti,ab,kw

#16 (arthralgia and temporomandibular):ti,ab,kw

#17 mandibular condyle$:ti,ab,kw

#18 temporal bone$:ti,ab,kw

#19 mandibular disease$:ti,ab,kw

#20 mandibular neoplasm$:ti,ab,kw

#21 prognathism:ti,ab,kw

#22 retrognathism:ti,ab,kw

#23 masticatory muscle pain:ti,ab,kw

#24 internal derangement:ti,ab,kw

#25 myofacial pain:ti,ab,kw

#26 (pain dysfunction syndrome or pds):ti,ab,kw

#27 myogenous pain:ti,ab,kw

#28 arthrogenous:ti,ab,kw

#29 localized myalgia:ti,ab,kw

#30 crepitation:ti,ab,kw

#31 costen$:ti,ab,kw

#32 (tooth near/5 grind$):ti,ab,kw

#33 (teeth near/5 grind$):ti,ab,kw

#34 (jaw near/5 clench$):ti,ab,kw

#35 (ear near/5 (click$ or pop$)):ti,ab,kw

#36 (jaw near/5 lock$):ti,ab,kw

#37 #1 or #2 or #3 or #4 or #5 or #6 or #7 or #8 or #9 or #10 or #11 or #12 or #13 or #14 or #15 or #16 or #17 or #18 or #19 or #20 or #21 or #22 or #23 or #24 or #25 or #26 or #27 or #28 or #29 or #30 or #31 or #32 or #33 or #34 or #35 or #36

#38 MeSH descriptor: [Bee Venoms] explode all trees

#39 pharmacopuncture:ti,ab,kw

#40 bee venom$:ti,ab,kw

#41 aquapuncture:ti,ab,kw

#42 aqua acupuncture:ti,ab,kw

#43 herbal acupuncture:ti,ab,kw

#44 point injection:ti,ab,kw

#45 hydroacupuncture:ti,ab,kw

#46 bee venom acupuncture:ti,ab,kw

#47 [mh "AcupuncturePoints"]

#48 [mh Injections]

#49 [mh "TriggerPoints"]

#50 acupoint*:ti,ab,kw

#51 injection*:ti,ab,kw

#52 #47 or #48 or #50

#53 #48 or #51

#54 #52 and #53

#55 #38 or #39 or #40 or #41 or #42 or #43 or #44 or #45 or #46 or #54

#56 #37 and #55

* Ovid-AMED

1 exp Temporomandibular joint/

2 exp Temporomandibular joint disease/

3 exp Mandibular disease/

4 exp Jaw/

5 exp Temporomandibular joint syndrome/

6 temporomandibular.mp.

7 temporo mandibular.mp.

8 (TMJ or TMD or CMD).mp.

9 (craniomandibular disorder$ or cranio mandibular).mp.

10 masticatory muscle$.mp.

11 masseter muscle$.mp.

12 pterygoid muscle$.mp.

13 temporal muscle$.mp.

14 (arthralgia and temporomandibular).mp.

15 (myofascial pain or mpd).mp.

16 mandibular condyle$.mp.

17 temporal bone$.mp.

18 mandibular disease$.mp.

19 mandibular neoplasm$.mp.

20 prognathism.mp.

21 retrognathism.mp.

22 masticatory muscle pain.mp.

23 internal derangement.mp.

24 myofacial pain.mp.

25 (pain dysfunction syndrome or pds).mp.

26 myogenous pain.mp.

27 arthrogenous.mp.

28 localized myalgia.mp.

29 crepitation.mp.

30 costen$.mp.

31 (tooth adj5 grind$).mp.

32 (teeth adj5 grind$).mp.

33 (jaw adj5 clench$).mp.

34 (ear adj5 (click$ or pop$)).mp.

35 (jaw adj5 lock$).mp.

36 or/1-35

37 pharmacopuncture.mp.

38 bee venom acupuncture.mp.

39 exp Injections/

40 acupoint$.mp.

41 bee venom$.mp.

42 aquapuncture.mp.

43 aqua acupuncture.mp.

44 herbal acupuncture.mp.

45 point injection.mp.

46 hydro acupuncture.mp.

47 injection$.mp.

48 39 or 47

49 exp Venoms/

50 exp Acupoints/

51 40 or 50

52 48 and 51

53 37 or 38 or 41 or 42 or 43 or 44 or 45 or 46 or 49 or 52

54 36 and 53

**Eq. (B.3) Herbal medicine (Q9,10,11)**

* Ovid-Medline

1 exp Temporomandibular Joint/

2 exp Temporomandibular Joint Disorders/

3 (temporomandibular or temporo mandibular or temporo­ mandibular).mp.

4 (TMJ or TMD or CMD).mp.

5 (craniomandibular disorder$ or cranio mandibular or cranio­mandibular disease$).mp.

6 exp masticatory muscles/

7 Mandibular Condyle/

8 exp mandibular diseases/

9 exp Craniomandibular Disorders/

10 exp Temporomandibular Joint Dysfunction Syndrome/

11 Myofascial Pain Syndromes/

12 masticatory muscle$.mp.

13 masseter muscle$.mp.

14 pterygoid muscle$.mp.

15 temporal muscle$.mp.

16 (arthralgia and temporomandibular).mp.

17 (myofascial pain or mpd).mp.

18 mandibular condyle$.mp.

19 temporal bone$.mp.

20 mandibular disease$.mp.

21 mandibular neoplasm$.mp.

22 prognathism.mp.

23 retrognathism.mp.

24 masticatory muscle pain.mp.

25 internal derangement.mp.

26 myofacial pain.mp.

27 disc displacement.mp.

28 (pain dysfunction syndrome or pds).mp.

29 myogenous pain.mp.

30 arthrogenous.mp.

31 localized myalgia.mp.

32 crepitation.mp.

33 costen$.mp.

34 (tooth adj5 grind$).mp.

35 (teeth adj5 grind$).mp.

36 (jaw adj5 clench$).mp.

37 (ear adj5 (click$ or pop$)).mp.

38 (jaw adj5 lock$).mp

39 exp Jaw Diseases/

40 (or/1­38) or 39

41 exp Medicine, Herbal/

42 exp Plants, Medicinal/

43 exp Medicine, Traditional/

44 exp Drugs, Chinese Herbal/

45 phytomedicine.tw.

46 botanical.tw.

47 ((traditional or chinese or herbal) adj medicine).tw.

48 ((oriental or chinese) adj tradition$).tw.

49 herbal drugs.tw.

50 herbal medicine.tw.

51 herbal preparations.tw.

52 herbs medicinal.tw.

53 herbs preparations.tw.

54 plants extract.tw.

55 plants medicinal.tw.

56 traditional Chinese medicine.tw.

57 traditional medicine.tw.

58 kampo medicine.tw.

59 decoction.tw.

60 $tang.mp.

61 &san.mp.

62 (herbal and (remed* or extract* or preparation* or mixture* or medic*)).tw.

63 (phyto and (drug* or pharmaceutical* or therap* or treatment* or medic*)).tw.

64 (plant* and (preparation* or extract* or medic*)).tw.

65 (chinese and (herb* or plant* or medic* or drug* or formul* or prescri*)).tw.

66 or/41­65

67 40 and 66

* Ovid-EMBASE

1 exp temporomandibular joint/

2 exp temporomandibular joint disorder/

3 (temporomandibular or temporo mandibular or temporomandibular).mp.

4 (TMJ or TMD or CMD).mp.

5 (craniomandibular disorder$ or cranio mandibular or craniomandibular disease$).mp.

6 exp masticatory muscle/

7 exp mandible condyle/

8 exp jaw disease/

9 masticatory muscle$.mp.

10 masseter muscle$.mp.

11 pterygoid muscle$.mp.

12 temporal muscle$.mp.

13 temporal muscle$.mp.

14 (arthralgia and temporomandibular).mp.

15 (myofascial pain or mpd).mp.

16 mandibular condyle$.mp.

17 temporal bone$.mp.

18 mandibular disease$.mp.

19 mandibular neoplasm$.mp.

20 prognathism.mp.

21 retrognathism.mp.

22 masticatory muscle pain.mp.

23 internal derangement.mp.

24 myogenous pain.mp.

25 arthrogenous.mp.

26 localized myalgia.mp.

27 crepitation.mp.

28 costen$.mp.

29 (tooth adj5 grind$).mp.

30 (teeth adj5 grind$).mp.

31 (jaw adj5 clench$).mp.

32 (ear adj5 (click$ or pop$)).mp.

33 (jaw adj5 lock$).mp.

34 OR/1-33

35 exp herbal medicine/

36 exp medicinal plant/

37 exp Chinese medicine/

38 exp Chinese herb/

39 exp Chinese drug/

40 phytomedicine.tw.

41 botanical.tw.

42 ((traditional or chinese or herbal) adj medicine).tw.

43 ((oriental or chinese) adj tradition$).tw.

44 herbal drugs.tw.

45 herbal medicine.tw.

46 herbal preparations.tw.

47 herbs medicinal.tw.

48 herbs preparations.tw.

49 plants extract.tw.

50 plants medicinal.tw.

51 traditional Chinese medicine.tw.

52 traditional medicine.tw.

53 kampo medicine.tw.

54 decoction.tw.

55 $tang.mp.

56 &san.mp.

57 (herbal and (remed* or extract* or preparation* or mixture* or medic*)).tw.

58 (phyto and (drug* or pharmaceutical* or therap* or treatment* or medic*)).tw.

59 (plant* and (preparation* or extract* or medic*)).tw.

60 (chinese and (herb* or plant* or medic* or drug* or formul* or prescri*)).tw.

61 or/35-60

62 34 and 61

* Ovid-AMED

1 exp Temporomandibular joint/

2 exp Temporomandibular joint disease/

3 exp Mandibular disease/

4 exp Jaw/

5 exp Temporomandibular joint syndrome/

6 temporomandibular.mp.

7 temporo mandibular.mp.

8 (TMJ or TMD or CMD).mp.

9 (craniomandibular disorder$ or cranio mandibular).mp.

10 masticatory muscle$.mp.

11 masseter muscle$.mp.

12 pterygoid muscle$.mp.

13 temporal muscle$.mp.

14 (arthralgia and temporomandibular).mp.

15 (myofascial pain or mpd).mp.

16 mandibular condyle$.mp.

17 temporal bone$.mp.

18 mandibular disease$.mp.

19 mandibular neoplasm$.mp.

20 prognathism.mp.

21 retrognathism.mp.

22 masticatory muscle pain.mp.

23 internal derangement.mp.

24 myofacial pain.mp.

25 (pain dysfunction syndrome or pds).mp.

26 myogenous pain.mp.

27 arthrogenous.mp.

28 localized myalgia.mp.

29 crepitation.mp.

30 costen$.mp.

31 (tooth adj5 grind$).mp.

32 (teeth adj5 grind$).mp.

33 (jaw adj5 clench$).mp.

34 (ear adj5 (click$ or pop$)).mp.

35 (jaw adj5 lock$).mp.

36 or/1-35

37 exp Herbs/

38 exp plant extracts/

39 exp plants medicinal/

40 exp traditional medicine chinese/

41 exp drugs chinese herbal/

42 phytomedicine.tw.

43 botanical.tw.

44 ((traditional or chinese or herbal) adj medicine).tw.

45 ((oriental or chinese) adj tradition$).tw.

46 herbal drugs.tw.

47 herbal medicine.tw.

48 herbal preparations.tw.

49 herbs medicinal.tw.

50 herbs preparations.tw.

51 plants extract.tw.

52 plants medicinal.tw.

53 traditional Chinese medicine.tw.

54 traditional medicine.tw.

55 kampo medicine.tw.

56 decoction.tw.

57 $tang.mp.

58 &san.mp.

59 (herbal and (remed* or extract* or preparation* or mixture* or medic*)).tw.

60 (phyto and (drug* or pharmaceutical* or therap* or treatment* or medic*)).tw.

61 (plant* and (preparation* or extract* or medic*)).tw.

62 (chinese and (herb* or plant* or medic* or drug* or formul* or prescri*)).tw.

63 or/37-62

64 36 and 63

* Cochrane Library

#1 MeSH descriptor: [Temporomandibular Joint] explode all trees

#2 MeSH descriptor: [Temporomandibular Joint Disorders] explode all trees

#3 MeSH descriptor: [Mandibular Condyle] explode all trees

#4 MeSH descriptor: [Masticatory Muscles] explode all trees

#5 MeSH descriptor: [Mandibular Diseases] explode all trees

#6 MeSH descriptor: [Craniomandibular Disorders] explode all trees

#7 MeSH descriptor: [Temporomandibular Joint Dysfunction Syndrome] explode all trees

#8 MeSH descriptor: [Jaw Diseases] explode all trees

#9 (temporomandibular or temporo mandibular or temporo­mandibular):ti,ab,kw

#10 (TMJ or TMD or CMD):ti,ab,kw

#11 (craniomandibular disorder$ or craniomandibular or cranio­mandibular disease$):ti,ab,kw (Word variations have been searched)

#12 masticatory muscle$:ti,ab,kw

#13 masseter muscle?:ti,ab,kw

#14 pterygoid muscle?:ti,ab,kw

#15 temporal muscle?:ti,ab,kw

#16 (arthralgia and temporomandibular):ti,ab,kw

#17 mandibular condyle$:ti,ab,kw

#18 temporal bone$:ti,ab,kw

#19 mandibular disease$:ti,ab,kw

#20 mandibular neoplasm$:ti,ab,kw

#21 prognathism:ti,ab,kw

#22 retrognathism:ti,ab,kw

#23 masticatory muscle pain:ti,ab,kw

#24 internal derangement:ti,ab,kw

#25 myofacial pain:ti,ab,kw

#26 (pain dysfunction syndrome or pds):ti,ab,kw

#27 myogenous pain:ti,ab,kw

#28 arthrogenous:ti,ab,kw

#29 localized myalgia:ti,ab,kw

#30 crepitation:ti,ab,kw

#31 costen$:ti,ab,kw

#32 (tooth near/5 grind$):ti,ab,kw

#33 (teeth near/5 grind$):ti,ab,kw

#34 (jaw near/5 clench$):ti,ab,kw

#35 (ear near/5 (click$ or pop$)):ti,ab,kw

#36 (jaw near/5 lock$):ti,ab,kw

#37 #1 or #2 or #3 or #4 or #5 or #6 or #7 or #8 or #9 or #10 or #11 or #12 or #13 or #14 or #15 or #16 or #17 or #18 or #19 or #20 or #21 or #22 or #23 or #24 or #25 or #26 or #27 or #28 or #29 or #30 or #31 or #32 or #33 or #34 or #35 or #36

#38 MeSH descriptor: [Herbal Medicine] explode all trees

#39 MeSH descriptor: [Medicine, Chinese Traditional] explode all trees

#40 MeSH descriptor: [Plants, Medicinal] explode all trees

#41 MeSH descriptor: [Drugs, Chinese Herbal] explode all trees

#42 phytomedicine:ti,ab,kw

#43 botanical:ti,ab,kw

#44 herbal drugs:ti,ab,kw

#45 herbal medicine:ti,ab,kw

#46 herbal preparations:ti,ab,kw

#47 herbs medicinal:ti,ab,kw

#48 herbs preparations:ti,ab,kw

#49 plants extract:ti,ab,kw

#50 plants medicinal:ti,ab,kw

#51 traditional Chinese medicine:ti,ab,kw

#52 traditional medicine:ti,ab,kw

#53 kampo medicine:ti,ab,kw

#54 decoction:ti,ab,kw

#55 *tang:ti,ab,kw

#56 *san:ti,ab,kw

#57 (herbal and (remed* or extract* or preparation* or mixture* or medic*)):ti,ab,kw

#58 (phyto and (drug* or pharmaceutical* or therap* or treatment* or medic*)):ti,ab,kw

#59 (plant* and (preparation* or extract* or medic*)):ti,ab,kw

#60 (chinese and (herb* or plant* or medic* or drug* or formul* or prescri*)):ti,ab,kw

#61 #26 or #27 or #28 or #29 or #30 or #31 or #32 or #33 or #34 or #35 or #36 or #37 or #38 or #39 or #40 or #41 or #42 or #43 or #44 or #45 or #46 or #47 or #48

**Eq. (B.4) Chuna manual therapy (Q6, 7, 8)**

* Ovid-Medline

1 exp Temporomandibular Joint/

2 exp Temporomandibular Joint Disorders/

3 (temporomandibular or temporo mandibular or temporo­mandibular).mp.

4 (TMJ or TMD or CMD).mp.

5 (craniomandibular disorder$ or cranio mandibular or cranio­mandibular disease$).mp.

6 exp masticatory muscles/

7 Mandibular Condyle/

8 exp mandibular diseases/

9 exp Craniomandibular Disorders/

10 exp Temporomandibular Joint Dysfunction Syndrome/

11 Myofascial Pain Syndromes/

12 masticatory muscle$.mp.

13 masseter muscle$.mp.

14 pterygoid muscle$.mp.

15 temporal muscle$.mp.

16 (arthralgia and temporomandibular).mp.

17 (myofascial pain or mpd).mp.

18 mandibular condyle$.mp.

19 temporal bone$.mp.

20 mandibular disease$.mp.

21 mandibular neoplasm$.mp.

22 prognathism.mp.

23 retrognathism.mp.

24 masticatory muscle pain.mp.

25 internal derangement.mp.

26 myofacial pain.mp.

27 disc displacement.mp.

28 (pain dysfunction syndrome or pds).mp.

29 myogenous pain.mp.

30 arthrogenous.mp.

31 localized myalgia.mp.

32 crepitation.mp.

33 costen$.mp.

34 (tooth adj5 grind$).mp.

35 (teeth adj5 grind$).mp.

36 (jaw adj5 clench$).mp.

37 (ear adj5 (click$ or pop$)).mp.

38 (jaw adj5 lock$).mp

39 exp Jaw Diseases/

40 (or/1­38) or 39

41 (Tui Na or Tuina).tw.

42 Chuna.tw.

43 (Chih Ya or Shiatsu or Shiatzu or Zhi Ya).tw.

44 41 or 42 or 43

45 40 AND 44

* Ovid-EMBASE

1 exp temporomandibular joint/

2 exp temporomandibular joint disorder/

3 (temporomandibular or temporo mandibular or temporomandibular).mp.

4 (TMJ or TMD or CMD).mp.

5 (craniomandibular disorder$ or cranio mandibular or craniomandibular disease$).mp.

6 exp masticatory muscle/

7 exp mandible condyle/

8 exp jaw disease/

9 masticatory muscle$.mp.

10 masseter muscle$.mp.

11 pterygoid muscle$.mp.

12 temporal muscle$.mp.

13 temporal muscle$.mp.

14 (arthralgia and temporomandibular).mp.

15 (myofascial pain or mpd).mp.

16 mandibular condyle$.mp.

17 temporal bone$.mp.

18 mandibular disease$.mp.

19 mandibular neoplasm$.mp.

20 prognathism.mp.

21 retrognathism.mp.

22 masticatory muscle pain.mp.

23 internal derangement.mp.

24 myogenous pain.mp.

25 arthrogenous.mp.

26 localized myalgia.mp.

27 crepitation.mp.

28 costen$.mp.

29 (tooth adj5 grind$).mp.

30 (teeth adj5 grind$).mp.

31 (jaw adj5 clench$).mp.

32 (ear adj5 (click$ or pop$)).mp.

33 (jaw adj5 lock$).mp.

34 OR/1-33

35 (Tui Na or Tuina).tw.

36 Chuna.tw.

37 35 or 36

38 34 and 37

* Ovid-AMED

1 exp Temporomandibular joint/

2 exp Temporomandibular joint disease/

3 exp Mandibular disease/

4 exp Jaw/

5 exp Temporomandibular joint syndrome/

6 temporomandibular.mp.

7 temporo mandibular.mp.

8 (TMJ or TMD or CMD).mp.

9 (craniomandibular disorder$ or cranio mandibular).mp.

10 masticatory muscle$.mp.

11 masseter muscle$.mp.

12 pterygoid muscle$.mp.

13 temporal muscle$.mp.

14 (arthralgia and temporomandibular).mp.

15 (myofascial pain or mpd).mp.

16 mandibular condyle$.mp.

17 temporal bone$.mp.

18 mandibular disease$.mp.

19 mandibular neoplasm$.mp.

20 prognathism.mp.

21 retrognathism.mp.

22 masticatory muscle pain.mp.

23 internal derangement.mp.

24 myofacial pain.mp.

25 (pain dysfunction syndrome or pds).mp.

26 myogenous pain.mp.

27 arthrogenous.mp.

28 localized myalgia.mp.

29 crepitation.mp.

30 costen$.mp.

31 (tooth adj5 grind$).mp.

32 (teeth adj5 grind$).mp.

33 (jaw adj5 clench$).mp.

34 (ear adj5 (click$ or pop$)).mp.

35 (jaw adj5 lock$).mp.

36 or/1-35

37 (Tui Na or Tuina).tw.

38 Chuna.tw.

39 (Chih Ya or Shiatsu or Shiatzu or Zhi Ya).tw.

40 37 or 38 or 39

41 36 AND 40

* Cochrane Library

#1 MeSH descriptor: [Temporomandibular Joint] explode all trees

#2 MeSH descriptor: [Temporomandibular Joint Disorders] explode all trees

#3 MeSH descriptor: [Mandibular Condyle] explode all trees

#4 MeSH descriptor: [Masticatory Muscles] explode all trees

#5 MeSH descriptor: [Mandibular Diseases] explode all trees

#6 MeSH descriptor: [Craniomandibular Disorders] explode all trees

#7 MeSH descriptor: [Temporomandibular Joint Dysfunction Syndrome] explode all trees

#8 MeSH descriptor: [Jaw Diseases] explode all trees

#9 (temporomandibular or temporo mandibular or temporo­mandibular):ti,ab,kw

#10 (TMJ or TMD or CMD):ti,ab,kw

#11 (craniomandibular disorder$ or craniomandibular or cranio­mandibular disease$):ti,ab,kw (Word variations have been searched)

#12 masticatory muscle$:ti,ab,kw

#13 masseter muscle?:ti,ab,kw

#14 pterygoid muscle?:ti,ab,kw

#15 temporal muscle?:ti,ab,kw

#16 (arthralgia and temporomandibular):ti,ab,kw

#17 mandibular condyle$:ti,ab,kw

#18 temporal bone$:ti,ab,kw

#19 mandibular disease$:ti,ab,kw

#20 mandibular neoplasm$:ti,ab,kw

#21 prognathism:ti,ab,kw

#22 retrognathism:ti,ab,kw

#23 masticatory muscle pain:ti,ab,kw

#24 internal derangement:ti,ab,kw

#25 myofacial pain:ti,ab,kw

#26 (pain dysfunction syndrome or pds):ti,ab,kw

#27 myogenous pain:ti,ab,kw

#28 arthrogenous:ti,ab,kw

#29 localized myalgia:ti,ab,kw

#30 crepitation:ti,ab,kw

#31 costen$:ti,ab,kw

#32 (tooth NEAR/5 grind$):ti,ab,kw

#33 (teeth NEAR/5 grind$):ti,ab,kw

#34 (jaw NEAR/5 clench$):ti,ab,kw

#35 (ear near/5 (click$ or pop$)):ti,ab,kw

#36 (jaw near/5 lock$):ti,ab,kw

#37 #1 or #2 or #3 or #4 or #5 or #6 or #7 or #8 or #9 or #10 or #11 or #12 or #13 or #14 or #15 or #16 or #17 or #18 or #19 or #20 or #21 or #22 or #23 or #24 or #25 or #26 or #27 or #28 or #29 or #30 or #31 or #32 or #33 or #34 or #35 or #36

#38 (Tui Na or Tuina):ti,ab,kw

#39 (chuna):ti,ab,kw

#40 (Chih Ya or Shiatsu or Shiatzu or Zhi Ya):ti,ab,kw

#41 #38 or #39 or #40

#42 #37 AND #41

**Eq. (B.5) Exercise (Q12)**

* Ovid-Medline

1 exp Temporomandibular Joint/

2 exp Temporomandibular Joint Disorders/

3 (temporomandibular or temporo mandibular or temporo­mandibular).mp.

4 (TMJ or TMD or CMD).mp.

5 (craniomandibular disorder$ or cranio mandibular or cranio­mandibular disease$).mp.

6 exp masticatory muscles/

7 Mandibular Condyle/

8 exp mandibular diseases/

9 exp Craniomandibular Disorders/

10 exp Temporomandibular Joint Dysfunction Syndrome/

11 Myofascial Pain Syndromes/

12 masticatory muscle$.mp.

13 masseter muscle$.mp.

14 pterygoid muscle$.mp.

15 temporal muscle$.mp.

16 (arthralgia and temporomandibular).mp.

17 (myofascial pain or mpd).mp.

18 mandibular condyle$.mp.

19 temporal bone$.mp.

20 mandibular disease$.mp.

21 mandibular neoplasm$.mp.

22 prognathism.mp.

23 retrognathism.mp.

24 masticatory muscle pain.mp.

25 internal derangement.mp.

26 myofacial pain.mp.

27 disc displacement.mp.

28 (pain dysfunction syndrome or pds).mp.

29 myogenous pain.mp.

30 arthrogenous.mp.

31 localized myalgia.mp.

32 crepitation.mp.

33 costen$.mp.

34 (tooth adj5 grind$).mp.

35 (teeth adj5 grind$).mp.

36 (jaw adj5 clench$).mp.

37 (ear adj5 (click$ or pop$)).mp.

38 (jaw adj5 lock$).mp

39 exp Jaw Diseases/

40 (or/1­38) or 39

41 exp Exercise/

42 exp Exercise Movement Techniques/

43 exercise.mp.

44 exp Patient Education as Topic/

45 exp Behavior Therapy/

46 patient education.mp.

47 behavior therapy.mp.

48 41 or 42 or 43 or 44 or 45 or 46 or 47

49 40 and 48

50 randomized controlled trial.pt.

51 controlled clinical trial.pt.

52 randomized.ab.

53 placebo.ab.

54 drug therapy.fs.

55 randomly.ab.

56 trial.ab.

57 groups.ab.

58 50 or 51 or 52 or 53 or 54 or 55 or 56 or 57

59 exp animals/ not humans.sh.

60 58 not 59

61 49 and 60

* Ovid-EMBASE

1 exp temporomandibular joint/

2 exp temporomandibular joint disorder/

3 (temporomandibular or temporo mandibular or temporomandibular).mp.

4 (TMJ or TMD or CMD).mp.

5 (craniomandibular disorder$ or cranio mandibular or craniomandibular disease$).mp.

6 exp masticatory muscle/

7 exp mandible condyle/

8 exp jaw disease/

9 masticatory muscle$.mp.

10 masseter muscle$.mp.

11 pterygoid muscle$.mp.

12 temporal muscle$.mp.

13 temporal muscle$.mp.

14 (arthralgia and temporomandibular).mp.

15 (myofascial pain or mpd).mp.

16 mandibular condyle$.mp.

17 temporal bone$.mp.

18 mandibular disease$.mp.

19 mandibular neoplasm$.mp.

20 prognathism.mp.

21 retrognathism.mp.

22 masticatory muscle pain.mp.

23 internal derangement.mp.

24 myogenous pain.mp.

25 arthrogenous.mp.

26 localized myalgia.mp.

27 crepitation.mp.

28 costen$.mp.

29 (tooth adj5 grind$).mp.

30 (teeth adj5 grind$).mp.

31 (jaw adj5 clench$).mp.

32 (ear adj5 (click$ or pop$)).mp.

33 (jaw adj5 lock$).mp.

34 OR/1-33

35 exp Exercise/

36 exp patient education/

37 exp behavior therapy/

38 exercise.mp.

39 patient education.mp.

40 behavior therapy.mp.

41 35 or 36 or 37 or 38 or 39 or 40

42 34 and 41

43 Clinical trial/

44 Randomized controlled trial/

45 Randomization/

46 Single blind procedure/

47 Double blind procedure/

48 Crossover procedure/

49 Placebo/

50 Randomi?ed controlled trial$.tw.

51 Rct.tw.

52 Random allocation.tw.

53 Randomly allocated.tw.

54 Allocated randomly.tw.

55 (allocated adj2 random).tw.

56 Single blind$.tw.

57 Double blind$.tw.

58 ((treble or triple) adj blind$).tw.

59 Placebo$.tw.

60 Prospective study/

61 or/43-60

62 42 and 61

* Ovid-AMED

1 exp Temporomandibular joint/

2 exp Temporomandibular joint disease/

3 exp Mandibular disease/

4 exp Jaw/

5 exp Temporomandibular joint syndrome/

6 temporomandibular.mp.

7 temporo mandibular.mp.

8 (TMJ or TMD or CMD).mp.

9 (craniomandibular disorder$ or cranio mandibular).mp.

10 masticatory muscle$.mp.

11 masseter muscle$.mp.

12 pterygoid muscle$.mp.

13 temporal muscle$.mp.

14 (arthralgia and temporomandibular).mp.

15 (myofascial pain or mpd).mp.

16 mandibular condyle$.mp.

17 temporal bone$.mp.

18 mandibular disease$.mp.

19 mandibular neoplasm$.mp.

20 prognathism.mp.

21 retrognathism.mp.

22 masticatory muscle pain.mp.

23 internal derangement.mp.

24 myofacial pain.mp.

25 (pain dysfunction syndrome or pds).mp.

26 myogenous pain.mp.

27 arthrogenous.mp.

28 localized myalgia.mp.

29 crepitation.mp.

30 costen$.mp.

31 (tooth adj5 grind$).mp.

32 (teeth adj5 grind$).mp.

33 (jaw adj5 clench$).mp.

34 (ear adj5 (click$ or pop$)).mp.

35 (jaw adj5 lock$).mp.

36 or/1-35

37 exp Exercise/

38 exercise.mp.

39 exp Patient education/

40 exp Behavior therapy/

41 patient education.mp.

42 behavior therapy.mp.

43 or/37-42

44 36 and 43

* Cochrane Library

#1 MeSH descriptor: [Temporomandibular Joint] explode all trees

#2 MeSH descriptor: [Temporomandibular Joint Disorders] explode all trees

#3 MeSH descriptor: [Mandibular Condyle] explode all trees

#4 MeSH descriptor: [Masticatory Muscles] explode all trees

#5 MeSH descriptor: [Mandibular Diseases] explode all trees

#6 MeSH descriptor: [Craniomandibular Disorders] explode all trees

#7 MeSH descriptor: [Temporomandibular Joint Dysfunction Syndrome] explode all trees

#8 MeSH descriptor: [Jaw Diseases] explode all trees

#9 (temporomandibular or temporo mandibular or temporo­mandibular):ti,ab,kw

#10 (TMJ or TMD or CMD):ti,ab,kw

#11 (craniomandibular disorder$ or craniomandibular or cranio­mandibular disease$):ti,ab,kw (Word variations have been searched)

#12 masticatory muscle$:ti,ab,kw

#13 masseter muscle?:ti,ab,kw

#14 pterygoid muscle?:ti,ab,kw

#15 temporal muscle?:ti,ab,kw

#16 (arthralgia and temporomandibular):ti,ab,kw

#17 mandibular condyle$:ti,ab,kw

#18 temporal bone$:ti,ab,kw

#19 mandibular disease$:ti,ab,kw

#20 mandibular neoplasm$:ti,ab,kw

#21 prognathism:ti,ab,kw

#22 retrognathism:ti,ab,kw

#23 masticatory muscle pain:ti,ab,kw

#24 internal derangement:ti,ab,kw

#25 myofacial pain:ti,ab,kw

#26 (pain dysfunction syndrome or pds):ti,ab,kw

#27 myogenous pain:ti,ab,kw

#28 arthrogenous:ti,ab,kw

#29 localized myalgia:ti,ab,kw

#30 crepitation:ti,ab,kw

#31 costen$:ti,ab,kw

#32 (tooth NEAR/5 grind$):ti,ab,kw

#33 (teeth NEAR/5 grind$):ti,ab,kw

#34 (jaw NEAR/5 clench$):ti,ab,kw

#35 (ear near/5 (click$ or pop$)):ti,ab,kw

#36 (jaw near/5 lock$):ti,ab,kw

#37 #1 or #2 or #3 or #4 or #5 or #6 or #7 or #8 or #9 or #10 or #11 or #12 or #13 or #14 or #15 or #16 or #17 or #18 or #19 or #20 or #21 or #22 or #23 or #24 or #25 or #26 or #27 or #28 or #29 or #30 or #31 or #32 or #33 or #34 or #35 or #36

#38 MeSH descriptor: [Exercise] explode all trees

#39 MeSH descriptor: [Exercise Movement Techniques] explode all trees

#40 MeSH descriptor: [Patient Education as Topic] explode all trees

#41 MeSH descriptor: [Behavior Therapy] explode all trees

#42 (exercise):ti,ab,kw

#43 (patient education):ti,ab,kw

#44 (behavior therapy):ti,ab,kw

#45 #38 or #39 or #40 or #41 or #42 or #43 or #44

#46 #37 AND #45

**Eq. (B.6) Occlusal splints (Q13)**

* Ovid-Medline

1 exp Temporomandibular Joint/

2 exp Temporomandibular Joint Disorders/

3 (temporomandibular or temporo mandibular or temporo­mandibular).mp.

4 (TMJ or TMD or CMD).mp.

5 (craniomandibular disorder$ or cranio mandibular or cranio­mandibular disease$).mp.

6 exp masticatory muscles/

7 Mandibular Condyle/

8 exp mandibular diseases/

9 exp Craniomandibular Disorders/

10 exp Temporomandibular Joint Dysfunction Syndrome/

11 Myofascial Pain Syndromes/

12 masticatory muscle$.mp.

13 masseter muscle$.mp.

14 pterygoid muscle$.mp.

15 temporal muscle$.mp.

16 (arthralgia and temporomandibular).mp.

17 (myofascial pain or mpd).mp.

18 mandibular condyle$.mp.

19 temporal bone$.mp.

20 mandibular disease$.mp.

21 mandibular neoplasm$.mp.

22 prognathism.mp.

23 retrognathism.mp.

24 masticatory muscle pain.mp.

25 internal derangement.mp.

26 myofacial pain.mp.

27 disc displacement.mp.

28 (pain dysfunction syndrome or pds).mp.

29 myogenous pain.mp.

30 arthrogenous.mp.

31 localized myalgia.mp.

32 crepitation.mp.

33 costen$.mp.

34 (tooth adj5 grind$).mp.

35 (teeth adj5 grind$).mp.

36 (jaw adj5 clench$).mp.

37 (ear adj5 (click$ or pop$)).mp.

38 (jaw adj5 lock$).mp

39 exp Jaw Diseases/

40 (or/1­38) or 39

41 exp splints/

42 occlusal splint$.mp.

43 oral splint$.mp.

44 stabilisation splint$.mp.

45 stabilization splint$.mp.

46 bite plate$.mp.

47 exp orthodontic appliances/

48 splint$.mp.

49 orthodontic appliance$.mp.

50 occlusal splint$.mp.

51 orthodontic Bracket$.mp.

52 orthodontic retainer$.mp.

53 orthodontic Wire$.mp.

54 orthopedic appliance$.mp.

55 (stabilization adj5 orthopedic).mp.

56 occlusal stabilization$.mp.

57 (stabilization adj5 bite adj5 splint$).mp.

58 stabilization splint$.mp.

59 muscle deprograming device$.mp.

60 gelb appliance$.mp.

61 tmj appliance$.mp.

62 tanner appliance$.mp.

63 (anterior adj5 repositioning adj5 appliance$).mp.

64 michigan splint$.mp.

65 (orthopedic adj5 stabilization).mp.

66 OR/41-65

67 40 AND 66

68 randomized controlled trial.pt.

69 controlled clinical trial.pt.

70 randomized.ab.

71 placebo.ab.

72 drug therapy.fs.

73 randomly.ab.

74 trial.ab.

75 groups.ab.

76 or/68-75

77 exp animals/ not humans.sh.

78 76 not 77

79 67 and 78

* Ovid-EMBASE

1 exp temporomandibular joint/

2 exp temporomandibular joint disorder/

3 (temporomandibular or temporo mandibular or temporomandibular).mp.

4 (TMJ or TMD or CMD).mp.

5 (craniomandibular disorder$ or cranio mandibular or craniomandibular disease$).mp.

6 exp masticatory muscle/

7 exp mandible condyle/

8 exp jaw disease/

9 masticatory muscle$.mp.

10 masseter muscle$.mp.

11 pterygoid muscle$.mp.

12 temporal muscle$.mp.

13 temporal muscle$.mp.

14 (arthralgia and temporomandibular).mp.

15 (myofascial pain or mpd).mp.

16 mandibular condyle$.mp.

17 temporal bone$.mp.

18 mandibular disease$.mp.

19 mandibular neoplasm$.mp.

20 prognathism.mp.

21 retrognathism.mp.

22 masticatory muscle pain.mp.

23 internal derangement.mp.

24 myogenous pain.mp.

25 arthrogenous.mp.

26 localized myalgia.mp.

27 crepitation.mp.

28 costen$.mp.

29 (tooth adj5 grind$).mp.

30 (teeth adj5 grind$).mp.

31 (jaw adj5 clench$).mp.

32 (ear adj5 (click$ or pop$)).mp.

33 (jaw adj5 lock$).mp.

34 OR/1-33

35 Clinical trial/

36 Randomized controlled trial/

37 Randomization/

38 Single blind procedure/

39 Double blind procedure/

40 Crossover procedure/

41 Placebo/

42 Randomi?ed controlled trial$.tw.

43 Rct.tw.

44 Random allocation.tw.

45 Randomly allocated.tw.

46 Allocated randomly.tw.

47 (allocated adj2 random).tw.

48 Single blind$.tw.

49 Double blind$.tw.

50 ((treble or triple) adj blind$).tw.

51 Placebo$.tw.

52 Prospective study/

53 or/35-52

54 exp splint/ or exp dental splint/

55 occlusal splint$.mp.

56 oral splint$.mp.

57 stabilisation splint$.mp.

58 stabilization splint$.mp.

59 bite plate$.mp.

60 splint$.mp.

61 orthodontic appliance$.mp.

62 orthodontic Bracket$.mp.

63 exp orthodontic device/

64 orthodontic retainer$.mp.

65 orthodontic Wire$.mp.

66 orthopedic appliance$.mp.

67 (stabilization adj5 orthopedic).mp.

68 occlusal stabilization$.mp.

69 (stabilization adj5 bite adj5 splint$).mp.

70 stabilization splint$.mp.

71 muscle deprograming device$.mp.

72 gelb appliance$.mp.

73 tmj appliance$.mp.

74 tanner appliance$.mp.

75 (anterior adj5 repositioning adj5 appliance$).mp.

76 michigan splint$.mp.

77 (orthopedic adj5 stabilization).mp.

78 or/54-77

79 34 and 53 and 78

* Ovid-AMED

1 exp Temporomandibular joint/

2 exp Temporomandibular joint disease/

3 exp Mandibular disease/

4 exp Jaw/

5 exp Temporomandibular joint syndrome/

6 temporomandibular.mp.

7 temporo mandibular.mp.

8 (TMJ or TMD or CMD).mp.

9 (craniomandibular disorder$ or cranio mandibular).mp.

10 masticatory muscle$.mp.

11 masseter muscle$.mp.

12 pterygoid muscle$.mp.

13 temporal muscle$.mp.

14 (arthralgia and temporomandibular).mp.

15 (myofascial pain or mpd).mp.

16 mandibular condyle$.mp.

17 temporal bone$.mp.

18 mandibular disease$.mp.

19 mandibular neoplasm$.mp.

20 prognathism.mp.

21 retrognathism.mp.

22 masticatory muscle pain.mp.

23 internal derangement.mp.

24 myofacial pain.mp.

25 (pain dysfunction syndrome or pds).mp.

26 myogenous pain.mp.

27 arthrogenous.mp.

28 localized myalgia.mp.

29 crepitation.mp.

30 costen$.mp.

31 (tooth adj5 grind$).mp.

32 (teeth adj5 grind$).mp.

33 (jaw adj5 clench$).mp.

34 (ear adj5 (click$ or pop$)).mp.

35 (jaw adj5 lock$).mp.

36 or/1-35

37 exp Splints/

38 occlusal splint$.mp.

39 oral splint$.mp.

40 stabilisation splint$.mp.

41 stabilization splint$.mp.

42 bite plate$.mp.

43 splint$.mp.

44 orthodontic appliance$.mp.

45 occlusal splint$.mp.

46 orthodontic Bracket$.mp.

47 orthodontic retainer$.mp.

48 orthodontic Wire$.mp.

49 orthopedic appliance$.mp.

50 (stabilization adj5 orthopedic).mp.

51 occlusal stabilization$.mp.

52 (stabilization adj5 bite adj5 splint$).mp.

53 stabilization splint$.mp.

54 muscle deprograming device$.mp.

55 gelb appliance$.mp.

56 tmj appliance$.mp.

57 tanner appliance$.mp.

58 (anterior adj5 repositioning adj5 appliance$).mp.

59 michigan splint$.mp.

60 (orthopedic adj5 stabilization).mp.

61 OR/37-60

62 36 AND 61

* Cochrane Library

#1 MeSH descriptor: [Temporomandibular Joint] explode all trees

#2 MeSH descriptor: [Temporomandibular Joint Disorders] explode all trees

#3 MeSH descriptor: [Mandibular Condyle] explode all trees

#4 MeSH descriptor: [Masticatory Muscles] explode all trees

#5 MeSH descriptor: [Mandibular Diseases] explode all trees

#6 MeSH descriptor: [Craniomandibular Disorders] explode all trees

#7 MeSH descriptor: [Temporomandibular Joint Dysfunction Syndrome] explode all trees

#8 MeSH descriptor: [Jaw Diseases] explode all trees

#9 (temporomandibular or temporo mandibular or temporo­mandibular):ti,ab,kw

#10 (TMJ or TMD or CMD):ti,ab,kw

#11 (craniomandibular disorder$ or craniomandibular or cranio­mandibular disease$):ti,ab,kw (Word variations have been searched)

#12 masticatory muscle$:ti,ab,kw

#13 masseter muscle?:ti,ab,kw

#14 pterygoid muscle?:ti,ab,kw

#15 temporal muscle?:ti,ab,kw

#16 (arthralgia and temporomandibular):ti,ab,kw

#17 mandibular condyle$:ti,ab,kw

#18 temporal bone$:ti,ab,kw

#19 mandibular disease$:ti,ab,kw

#20 mandibular neoplasm$:ti,ab,kw

#21 prognathism:ti,ab,kw

#22 retrognathism:ti,ab,kw

#23 masticatory muscle pain:ti,ab,kw

#24 internal derangement:ti,ab,kw

#25 myofacial pain:ti,ab,kw

#26 (pain dysfunction syndrome or pds):ti,ab,kw

#27 myogenous pain:ti,ab,kw

#28 arthrogenous:ti,ab,kw

#29 localized myalgia:ti,ab,kw

#30 crepitation:ti,ab,kw

#31 costen$:ti,ab,kw

#32 (tooth NEAR/5 grind$):ti,ab,kw

#33 (teeth NEAR/5 grind$):ti,ab,kw

#34 (jaw NEAR/5 clench$):ti,ab,kw

#35 (ear near/5 (click$ or pop$)):ti,ab,kw

#36 (jaw near/5 lock$):ti,ab,kw

#37 #1 or #2 or #3 or #4 or #5 or #6 or #7 or #8 or #9 or #10 or #11 or #12 or #13 or #14 or #15 or #16 or #17 or #18 or #19 or #20 or #21 or #22 or #23 or #24 or #25 or #26 or #27 or #28 or #29 or #30 or #31 or #32 or #33 or #34 or #35 or #36

#38 MeSH descriptor: [Splints] explode all trees

#39 MeSH descriptor: [Orthodontic Appliances] explode all trees

#40 (occlusal splint$):ti,ab,kw

#41 (oral splint$):ti,ab,kw

#42 (stabilisation splint$):ti,ab,kw

#43 (stabilization splint$):ti,ab,kw

#44 (biteplate$):ti,ab,kw

#45 (splint$):ti,ab,kw

#46 (orthodontic appliance$):ti,ab,kw

#47 (occlusal splint$):ti,ab,kw

#48 (orthodontic Bracket$):ti,ab,kw

#49 (orrthodontic retainer$):ti,ab,kw

#50 (orthodontic Wire$):ti,ab,kw

#51 orthodontic appliance$:ti,ab,kw

#52 (stabilization near/5 orthopedic):ti,ab,kw

#53 occlusal stabilization$:ti,ab,kw

#54 (stabilization near/5 bite near/5 splint$):ti,ab,kw

#55 stabilization splint$:ti,ab,kw

#56 muscle deprograming device$:ti,ab,kw

#57 gelb appliance$:ti,ab,kw

#58 tmj appliance:ti,ab,kw

#59 tanner appliance:ti,ab,kw

#60 (anterior near/5 repositioning near/5 appliance$):ti,ab,kw

#61 michigan splint$:ti,ab,kw

#62 (orthopedic near/5 stabilization):ti,ab,kw

#63 #38 or #39 or #40 or #41 or #42 or #43 or #44 or #45 or #46 or #47 or #48 or #49 or #50 or #51 or #52 or #53 or #54 or #55 or #56 or #57 or #58 or #59 or #60 or #61 or #62

#64 #37 and #63

Eq. (B.7) Mae-sun (Q14)

* Ovid-Medline

1 exp Temporomandibular Joint/

2 exp Temporomandibular Joint Disorders/

3 (temporomandibular or temporo mandibular or temporo­mandibular).mp.

4 (TMJ or TMD or CMD).mp.

5 (craniomandibular disorder$ or cranio mandibular or cranio­mandibular disease$).mp.

6 exp masticatory muscles/

7 Mandibular Condyle/

8 exp mandibular diseases/

9 exp Craniomandibular Disorders/

10 exp Temporomandibular Joint Dysfunction Syndrome/

11 Myofascial Pain Syndromes/

12 masticatory muscle$.mp.

13 masseter muscle$.mp.

14 pterygoid muscle$.mp.

15 temporal muscle$.mp.

16 (arthralgia and temporomandibular).mp.

17 (myofascial pain or mpd).mp.

18 mandibular condyle$.mp.

19 temporal bone$.mp.

20 mandibular disease$.mp.

21 mandibular neoplasm$.mp.

22 prognathism.mp.

23 retrognathism.mp.

24 masticatory muscle pain.mp.

25 internal derangement.mp.

26 myofacial pain.mp.

27 disc displacement.mp.

28 (pain dysfunction syndrome or pds).mp.

29 myogenous pain.mp.

30 arthrogenous.mp.

31 localized myalgia.mp.

32 crepitation.mp.

33 costen$.mp.

34 (tooth adj5 grind$).mp.

35 (teeth adj5 grind$).mp.

36 (jaw adj5 clench$).mp.

37 (ear adj5 (click$ or pop$)).mp.

38 (jaw adj5 lock$).mp

39 exp Jaw Diseases/

40 (or/1­38) or 39

41 exp Catgut/

42 catgut implantation.tw.

43 catgut emb*.tw.

44 thread emb*.tw.

45 thread implantation.tw.

46 or/41-45

47 40 and 46

* Ovid-EMBASE

1 exp temporomandibular joint/

2 exp temporomandibular joint disorder/

3 (temporomandibular or temporo mandibular or temporomandibular).mp.

4 (TMJ or TMD or CMD).mp.

5 (craniomandibular disorder$ or cranio mandibular or craniomandibular disease$).mp.

6 exp masticatory muscle/

7 exp mandible condyle/

8 exp jaw disease/

9 masticatory muscle$.mp.

10 masseter muscle$.mp.

11 pterygoid muscle$.mp.

12 temporal muscle$.mp.

13 temporal muscle$.mp.

14 (arthralgia and temporomandibular).mp.

15 (myofascial pain or mpd).mp.

16 mandibular condyle$.mp.

17 temporal bone$.mp.

18 mandibular disease$.mp.

19 mandibular neoplasm$.mp.

20 prognathism.mp.

21 retrognathism.mp.

22 masticatory muscle pain.mp.

23 internal derangement.mp.

24 myogenous pain.mp.

25 arthrogenous.mp.

26 localized myalgia.mp.

27 crepitation.mp.

28 costen$.mp.

29 (tooth adj5 grind$).mp.

30 (teeth adj5 grind$).mp.

31 (jaw adj5 clench$).mp.

32 (ear adj5 (click$ or pop$)).mp.

33 (jaw adj5 lock$).mp.

34 OR/1-33

35 exp Catgut/

36 catgut implantation.tw.

37 catgut emb*.tw.

38 thread emb*.tw.

39 thread implantation.tw.

40 35 or 36 or 37 or 38 or 39

41 34 and 40

* Ovid-AMED

1 exp Temporomandibular joint/

2 exp Temporomandibular joint disease/

3 exp Mandibular disease/

4 exp Jaw/

5 exp Temporomandibular joint syndrome/

6 temporomandibular.mp.

7 temporo mandibular.mp.

8 (TMJ or TMD or CMD).mp.

9 (craniomandibular disorder$ or cranio mandibular).mp.

10 masticatory muscle$.mp.

11 masseter muscle$.mp.

12 pterygoid muscle$.mp.

13 temporal muscle$.mp.

14 (arthralgia and temporomandibular).mp.

15 (myofascial pain or mpd).mp.

16 mandibular condyle$.mp.

17 temporal bone$.mp.

18 mandibular disease$.mp.

19 mandibular neoplasm$.mp.

20 prognathism.mp.

21 retrognathism.mp.

22 masticatory muscle pain.mp.

23 internal derangement.mp.

24 myofacial pain.mp.

25 (pain dysfunction syndrome or pds).mp.

26 myogenous pain.mp.

27 arthrogenous.mp.

28 localized myalgia.mp.

29 crepitation.mp.

30 costen$.mp.

31 (tooth adj5 grind$).mp.

32 (teeth adj5 grind$).mp.

33 (jaw adj5 clench$).mp.

34 (ear adj5 (click$ or pop$)).mp.

35 (jaw adj5 lock$).mp.

36 or/1-35

37 catgut implantation.tw.

38 catgut emb*.tw.

39 thread emb*.tw.

40 thread implantation.tw.

41 37 or 38 or 39 or 40

42 36 and 41

* Cochrane Library

#1 MeSH descriptor: [Temporomandibular Joint] explode all trees

#2 MeSH descriptor: [Temporomandibular Joint Disorders] explode all trees

#3 MeSH descriptor: [Mandibular Condyle] explode all trees

#4 MeSH descriptor: [Masticatory Muscles] explode all trees

#5 MeSH descriptor: [Mandibular Diseases] explode all trees

#6 MeSH descriptor: [Craniomandibular Disorders] explode all trees

#7 MeSH descriptor: [Temporomandibular Joint Dysfunction Syndrome] explode all trees

#8 MeSH descriptor: [Jaw Diseases] explode all trees

#9 (temporomandibular or temporo mandibular or temporo­mandibular):ti,ab,kw

#10 (TMJ or TMD or CMD):ti,ab,kw

#11 (craniomandibular disorder$ or craniomandibular or cranio­mandibular disease$):ti,ab,kw

#12 masticatory muscle$:ti,ab,kw

#13 masseter muscle?:ti,ab,kw

#14 pterygoid muscle?:ti,ab,kw

#15 temporal muscle?:ti,ab,kw

#16 (arthralgia and temporomandibular):ti,ab,kw

#17 mandibular condyle$:ti,ab,kw

#18 temporal bone$:ti,ab,kw

#19 mandibular disease$:ti,ab,kw

#20 mandibular neoplasm$:ti,ab,kw

#21 prognathism:ti,ab,kw

#22 retrognathism:ti,ab,kw

#23 masticatory muscle pain:ti,ab,kw

#24 internal derangement:ti,ab,kw

#25 myofacial pain:ti,ab,kw

#26 (pain dysfunction syndrome or pds):ti,ab,kw

#27 myogenous pain:ti,ab,kw

#28 arthrogenous:ti,ab,kw

#29 localized myalgia:ti,ab,kw

#30 crepitation:ti,ab,kw

#31 costen$:ti,ab,kw

#32 (tooth NEAR/5 grind$):ti,ab,kw

#33 (teeth NEAR/5 grind$):ti,ab,kw

#34 (jaw NEAR/5 clench$):ti,ab,kw

#35 (ear near/5 (click$ or pop$)):ti,ab,kw

#36 (jaw near/5 lock$):ti,ab,kw

#37 #1 or #2 or #3 or #4 or #5 or #6 or #7 or #8 or #9 or #10 or #11 or #12 or #13 or #14 or #15 or #16 or #17 or #18 or #19 or #20 or #21 or #22 or #23 or #24 or #25 or #26 or #27 or #28 or #29 or #30 or #31 or #32 or #33 or #34 or #35 or #36

#38 MeSH descriptor: [Catgut] explode all trees

#39 (catgut implantation):ti,ab,kw

#40 (catgut emb*):ti,ab,kw

#41 (thread emb*):ti,ab,kw

#42 (thread implantation):ti,ab,kw

#43 #38 or #39 or #40 or #41 or #42

#44 #37 and #43

**Eq. (B.8) Physical therapy (Q15-Q17)**

*** Ovid-Medline**

1 exp Temporomandibular Joint/

2 exp Temporomandibular Joint Disorders/

3 (temporomandibular or temporo mandibular or temporo­mandibular).mp.

4 (TMJ or TMD or CMD).mp.

5 (craniomandibular disorder$ or cranio mandibular or cranio­mandibular disease$).mp.

6 exp masticatory muscles/

7 Mandibular Condyle/

8 exp mandibular diseases/

9 exp Craniomandibular Disorders/

10 exp Temporomandibular Joint Dysfunction Syndrome/

11 Myofascial Pain Syndromes/

12 masticatory muscle$.mp.

13 masseter muscle$.mp.

14 pterygoid muscle$.mp.

15 temporal muscle$.mp.

16 (arthralgia and temporomandibular).mp.

17 (myofascial pain or mpd).mp.

18 mandibular condyle$.mp.

19 temporal bone$.mp.

20 mandibular disease$.mp.

21 mandibular neoplasm$.mp.

22 prognathism.mp.

23 retrognathism.mp.

24 masticatory muscle pain.mp.

25 internal derangement.mp.

26 myofacial pain.mp.

27 disc displacement.mp.

28 (pain dysfunction syndrome or pds).mp.

29 myogenous pain.mp.

30 arthrogenous.mp.

31 localized myalgia.mp.

32 crepitation.mp.

33 costen$.mp.

34 (tooth adj5 grind$).mp.

35 (teeth adj5 grind$).mp.

36 (jaw adj5 clench$).mp.

37 (ear adj5 (click$ or pop$)).mp.

38 (jaw adj5 lock$).mp.

39 exp Jaw Diseases/

40 (or/1­38) or 39

41 exp Physical Therapy Modalities/

42 physiotherapy.mp.

43 exp Lasers/

44 laser.mp.

45 TENS.mp. or exp Transcutaneous Electric Nerve Stimulation/

46 exp Ultrasonic Therapy/

47 or/41-46

48 40 and 47

49 randomized controlled trial.pt.

50 controlled clinical trial.pt.

51 randomized.ab.

52 placebo.ab.

53 drug therapy.fs.

54 randomly.ab.

55 trial.ab.

56 groups.ab.

57 or/49-56

58 exp animals/ not humans.sh.

59 57 not 58

60 48 and 59

* Ovid-EMBASE

1 exp Temporomandibular Joint/

2 exp Temporomandibular Joint Disorders/

3 (temporomandibular or temporo mandibular or temporo­mandibular).mp.

4 (TMJ or TMD or CMD).mp.

5 (craniomandibular disorder$ or cranio mandibular or cranio­mandibular disease$).mp.

6 exp masticatory muscles/

7 exp mandibular Condyle/

8 exp jaw disease/

9 masticatory muscle$.mp.

10 masseter muscle$.mp.

11 pterygoid muscle$.mp.

12 temporal muscle$.mp.

13 temporal muscle$.mp.

14 (arthralgia and temporomandibular).mp.

15 (myofascial pain or mpd).mp.

16 mandibular condyle$.mp.

17 temporal bone$.mp.

18 mandibular disease$.mp.

19 mandibular neoplasm$.mp.

20 prognathism.mp.

21 retrognathism.mp.

22 masticatory muscle pain.mp.

23 internal derangement.mp.

24 myogenous pain.mp.

25 arthrogenous.mp.

26 localized myalgia.mp.

27 crepitation.mp.

28 costen$.mp.

29 tooth adj5 grind$).mp.

30 teeth adj5 grind$).mp.

31 (jaw adj5 clench$).mp.

32 (ear adj5 (click$ or pop$)).mp.

33 (jaw adj5 lock$).mp.

34 or/1-33

35 Clinical trial/

36 Randomized controlled trial/

37 Randomization/

38 Single blind procedure/

39 Double blind procedure/

40 3Crossover procedure/

41 Placebo/

42 Randomi?ed controlled trial$.tw.

43 Rct.tw.

44 Random allocation.tw.

45 Randomly allocated.tw.

46 Allocated randomly.tw.

47 (allocated adj2 random).tw.

48 Single blind$.tw.

49 Double blind$.tw.

50 ((treble or triple) adj blind$).tw.

51 Placebo$.tw.

52 Prospective study/

53 OR/35-52

54 exp physiotherapy practice/ or exp physiotherapy/

55 exp laser/

56 physiotherapy.mp.

57 laser.mp.

58 exp transcutaneous electrical nerve stimulation/

59 exp ultrasound/

60 TENS.mp.

61 ultrasound.mp.

62 54 or 55 or 56 or 57 or 58 or 59 or 60 or 61

63 34 and 53 and 62

* Ovid-AMED

1 exp Temporomandibular joint/

2 exp Temporomandibular joint disease/

3 exp Mandibular disease/

4 exp Jaw/

5 exp Temporomandibular joint syndrome/

6 temporomandibular.mp.

7 temporo mandibular.mp.

8 (TMJ or TMD or CMD).mp.

9 (craniomandibular disorder$ or cranio mandibular).mp.

10 masticatory muscle$.mp.

11 masseter muscle$.mp.

12 pterygoid muscle$.mp.

13 temporal muscle$.mp.

14 (arthralgia and temporomandibular).mp.

15 (myofascial pain or mpd).mp.

16 mandibular condyle$.mp.

17 temporal bone$.mp.

18 mandibular disease$.mp.

19 mandibular neoplasm$.mp.

20 prognathism.mp.

21 retrognathism.mp.

22 masticatory muscle pain.mp.

23 internal derangement.mp.

24 myofacial pain.mp.

25 (pain dysfunction syndrome or pds).mp.

26 myogenous pain.mp.

27 arthrogenous.mp.

28 localized myalgia.mp.

29 crepitation.mp.

30 costen$.mp.

31 (tooth adj5 grind$).mp.

32 (teeth adj5 grind$).mp.

33 (jaw adj5 clench$).mp.

34 (ear adj5 (click$ or pop$)).mp.

35 (jaw adj5 lock$).mp.

36 or/1-35

37 exp physical therapy modalities/

38 physiotherapy.mp.

39 exp Lasers/

40 laser.mp.

41 exp Transcutaneous electric nerve stimulation/

42 TENS.mp.

43 exp Ultrasonic Therapy/

44 or/37-43

45 36 and 44

* cochrane

#1 MeSH descriptor: [Temporomandibular Joint] explode all trees

#2 MeSH descriptor: [Temporomandibular Joint Disorders] explode all trees

#3 MeSH descriptor: [Mandibular Condyle] explode all trees

#4 MeSH descriptor: [Masticatory Muscles] explode all trees

#5 MeSH descriptor: [Mandibular Diseases] explode all trees

#6 MeSH descriptor: [Craniomandibular Disorders] explode all trees

#7 MeSH descriptor: [Temporomandibular Joint Dysfunction Syndrome] explode all trees

#8 MeSH descriptor: [Jaw Diseases] explode all trees

#9 (temporomandibular or temporo mandibular or temporo­ mandibular):ti,ab,kw

#10 (TMJ or TMD or CMD):ti,ab,kw

#11 (craniomandibular disorder$ or cranio mandibular or cranio­mandibular disease$):ti,ab,kw (Word variations have been searched)

#12 masticatory muscle$:ti,ab,kw

#13 masseter muscle?:ti,ab,kw

#14 pterygoid muscle?:ti,ab,kw

#15 temporal muscle?:ti,ab,kw

#16 (arthralgia and temporomandibular):ti,ab,kw

#17 mandibular condyle$:ti,ab,kw

#18 temporal bone$:ti,ab,kw

#19 mandibular disease$:ti,ab,kw

#20 mandibular neoplasm$:ti,ab,kw

#21 prognathism:ti,ab,kw

#22 retrognathism:ti,ab,kw

#23 masticatory muscle pain:ti,ab,kw

#24 internal derangement:ti,ab,kw

#25 myofacial pain:ti,ab,kw

#26 (pain dysfunction syndrome or pds):ti,ab,kw

#27 myogenous pain:ti,ab,kw

#28 arthrogenous:ti,ab,kw

#29 localized myalgia:ti,ab,kw

#30 crepitation:ti,ab,kw

#31 costen$:ti,ab,kw

#32 (tooth near/5 grind$):ti,ab,kw

#33 (teeth near/5 grind$):ti,ab,kw

#34 (jaw near/5 clench$):ti,ab,kw

#35 (ear near/5 (click$ or pop$)):ti,ab,kw

#36 (jaw near/5 lock$):ti,ab,kw

#37 #1 or #2 or #3 or #4 or #5 or #6 or #7 or #8 or #9 or #10 or #11 or #12 or #13 or #14 or #15 or #16 or #17 or #18 or #19 or #20 or #21 or #22 or #23 or #24 or #25 or #26 or #27 or #28 or #29 or #30 or #31 or #32 or #33 or #34 or #35 or #36

#38 MeSH descriptor: [Physical Therapy Modalities] explode all trees

#39 MeSH descriptor: [Lasers] explode all trees

#40 MeSH descriptor: [Transcutaneous Electric Nerve Stimulation] explode all trees

#41 MeSH descriptor: [Ultrasonic Therapy] explode all trees

#42 (physiotherapy):ti,ab,kw

#43 (laser):ti,ab,kw

#44 (TENS):ti,ab,kw

#45 #38 or #39 or #40 or #41 or #42 or #43 or #44

#46 #37 AND #45

**Eq. (B.9) Korean, Chinese, and Japanese DB**

1. **Korean**

* OASIS

TMJ OR temporomandibular OR 턱관절장애 OR TMD OR CMD OR craniomandibular OR 턱관절

* NDSL

전체:(TMJ OR temporomandibular OR 턱관절장애 OR TMD OR CMD OR craniomandibular)

* KISS

TMJ OR temporomandibular OR TMD OR CMD OR craniomandibular

턱관절장애

* KoreaMed

TMJ OR temporomandibular OR TMD OR CMD OR craniomandibular

* KMBASE

TMJ OR temporomandibular OR 턱관절장애 OR TMD OR CMD OR craniomandibular

1. **Chinese**

* CNKI

1 (SU='颞下颌关节' or SU='颞下颌关节紊乱综合征'or SU='颞下颌关节紊乱病'or SU='下颌骨病变') AND (SU='电针' OR SU='针')

2 (SU='颞下颌关节' or SU='颞下颌关节紊乱综合征'or SU='颞下颌关节紊乱病'or SU='下颌骨病变') AND (SU='水针' OR SU='穴位封闭' OR SU='穴位注射' OR SU='acupoint injection' OR SU='蜂毒' OR SU='蜂针' OR SU='蜂')

3 (SU='颞下颌关节' or SU='颞下颌关节紊乱综合征'or SU='颞下颌关节紊乱病'or SU='下颌骨病变') AND (SU='中药' OR SU='herbal medicine' OR SU='丹' or SU='汤' or SU='丸' or SU='散')

4 ((SU='颞下颌关节' or SU='颞下颌关节紊乱综合征'or SU='颞下颌关节紊乱病'or SU='下颌骨病变') AND (SU='导引' OR SU='daoyin' OR SU='tuina' OR SU='推拿' OR SU='手法' ') AND (SU='随机' or SU='对照')

5 ((SU='颞下颌关节' or SU='颞下颌关节紊乱综合征'or SU='颞下颌关节紊乱病'or SU='下颌骨病变') AND (SU='咬合夹板' or SU='夹板' or SU='正畸' or SU='稳定型咬合板') AND (SU='随机')

6 (SU='颞下颌关节' or SU='颞下颌关节紊乱综合征'or SU='颞下颌关节紊乱病'or SU='下颌骨病变'or SU='temporomandibular') AND (SU='穴位埋线' or SU='catgut embedding' or SU='thread embedding' or SU='thread implant' or SU='catgut implant')

7 ((SU='颞下颌关节' or SU='颞下颌关节紊乱综合征'or SU='颞下颌关节紊乱病'or SU='下颌骨病变') AND (SU=‘运动疗法‘ SU='导引' or SU='daoyin') AND (SU='随机')

8 ((SU='颞下颌关节' or SU='颞下颌关节紊乱综合征'or SU='颞下颌关节紊乱病'or SU='下颌骨病变') AND (SU='热疗' OR SU='冷冻疗法' OR SU='颈痛' OR SU='电疗法' OR SU='物理治疗' OR SU='康复理疗') AND (SU='随机')

1. **Japanese**

* Citation Information by NII (Cinii)

1 (顎関節 OR 顎関節機能不全症候群 OR TMJ OR TMD OR temporomandibular OR craniomandibular) AND (鍼療法 OR 鍼灸医学 OR 電気鍼療法)

2 (顎関節 OR 顎関節機能不全症候群 OR TMJ OR TMD OR temporomandibular OR craniomandibular) AND (蜂毒 OR 蜂 OR acupoint injection OR Bee Venom Injection)

3 (顎関節 OR 顎関節機能不全症候群 OR TMJ OR TMD OR temporomandibular OR craniomandibular) AND (漢方医学 OR 漢方薬 OR KAMPO OR herbal medicine OR 丹 OR 湯 OR 丸 OR 散)

4 (顎関節 OR 顎関節機能不全症候群 OR TMJ OR TMD OR temporomandibular OR craniomandibular) AND (手法)

5 (顎関節 OR 顎関節機能不全症候群 OR TMJ OR TMD OR temporomandibular OR craniomandibular) AND (exercise OR exercise or exercise movement techniques OR 身体運動)

6 (顎関節 OR 顎関節機能不全症候群 OR TMJ OR TMD OR temporomandibular OR craniomandibular) AND (splint OR oral splint OR orthodontic appliances OR occlusal stabilization)

7 (顎関節 OR 顎関節機能不全症候群 OR TMJ OR TMD OR temporomandibular OR craniomandibular) AND (catgut embedding OR thread embedding OR thread implant OR catgut implant)

8 (顎関節 OR 顎関節機能不全症候群 OR TMJ OR TMD OR temporomandibular OR craniomandibular) AND (physical therapy)

**PRISMA flow chart**

**Eq. (C.1) Acupuncture and laser acupuncture (Q1-4)**

| **Identification** |  | Records identified through database searching  1) Medline (n=728),  2) EMBASE (n=711),  3) Cochrane(n=246),  4) AMED (n=375),  5) CNKI (n=90)  6) Cinii (n=1)  7) KoreaMed (n=1)  8) KMBASE (n=77)  9) OASIS (n=88)  10) NDSL (n=112)  11) KISS (n=87) | | |  | Additional records identified through other sources  (n=3) | | | |  | |
| --- | --- | --- | --- | --- | --- | --- | --- | --- | --- | --- | --- |
|  |  | ↓ | | |  | ↓ | | | |  | |
| **Screening** |  | Records after duplicates(n=130) removed (n=2389) | | | | | | | |  | |
|  |  |  | | ↓ | | |  | | |  | |
|  |  |  | Records screened  (n=2389) | | | | | → | Records excluded  (n=2199) | |  |
|  |  |  | | ↓ | | |  | | |  | |
| **Eligibility** |  |  | Full-text articles assessed for eligibility  (n=190) | | | | | → | Full-text articles excluded, with reasons  inconsistency with P : 10  inconsistency with I : 35  inconsistency with C : 8  inconsistency with O : 3  not RCTs : 85  duplication : 9 | |  |
|  |  |  | | ↓ | | |  | | |  | |
| **Included** |  |  | Studies included in qualitative synthesis  (Q1 n=14)  (Q2 n= 16)  (Q2-1 n=1)  (Q3 n= 5)  (Q4 n=4) | | | | |  |  |  | |

**Eq. (C.2) Pharmacopuncture (Q5)**

| **Identification** |  | Records identified through database searching  1) Medline (n=164),  2) EMBASE (n=319),  3) Cochrane(n=82),  4) AMED (n=14),  5) CNKI (n=26)  6) Cinii (n=10)  7) KoreaMed (n=1)  8) KMBASE (n=3)  9) OASIS (n=84)  10) NDSL (n=7)  11) KISS (n=4) | | |  | Additional records identified through other sources  (n=1) | | | |  | |
| --- | --- | --- | --- | --- | --- | --- | --- | --- | --- | --- | --- |
|  |  | ↓ | | |  | ↓ | | | |  | |
| **Screening** |  | Records after duplicates (n=554) removed  (n=162) | | | | | | | |  | |
|  |  |  | | ↓ | | |  | | |  | |
|  |  |  | Records screened  (n=554) | | | | | → | Records excluded  (n=528) | |  |
|  |  |  | | ↓ | | |  | | |  | |
| **Eligibility** |  |  | Full-text articles assessed for eligibility  (n=26) | | | | | → | Full-text articles excluded, with reasons  not intervention: 9  not comparison: 2  not RCT: 14 | |  |
|  |  |  | | ↓ | | |  | | |  | |
| **Included** |  |  | Studies included in qualitative synthesis  (Q5 n=1) | | | | |  |  |  | |

**Eq. (C.3) Chuna (Q6,7,8)**

| **Identification** |  | Records identified through database searching  1) Medline (n=16),  2) EMBASE (n=7),  3) Cochrane(n=9),  4) AMED (n=4),  5) CNKI (n=233)  6) Cinii (n=71)  7) KoreaMed (n=0)  8) KMBASE (n=20)  9) OASIS (n=89)  10) NDSL (n=33)  11) KISS (n=27) | | |  | Additional records identified through other sources  (n=0) | | | |  | |
| --- | --- | --- | --- | --- | --- | --- | --- | --- | --- | --- | --- |
|  |  | ↓ | | |  | ↓ | | | |  | |
| **Screening** |  | Records after duplicates(n=100) removed (n=409) | | | | | | | |  | |
|  |  |  | | ↓ | | |  | | |  | |
|  |  |  | Records screened  (n=409) | | | | | → | Records excluded  (n=313) | |  |
|  |  |  | | ↓ | | |  | | |  | |
| **Eligibility** |  |  | Full-text articles assessed for eligibility  (n=96) | | | | | → | Full-text articles excluded, with reasons  not participants: 1  not intervention: 22  not comparison: 6  not outcome: 1  not RCT or proceeding: 53  duplicate: 1 | |  |
|  |  |  | | ↓ | | |  | | |  | |
| **Included** |  |  | Studies included in qualitative synthesis  (Q6 n=5)  (Q7 n=2)  (Q8 n=5) | | | | |  |  |  | |

**Eq. (C.4) Herbal medicine (Q9-11)**

| **Identification** |  | Records identified through database searching  1) Medline (n=609),  2) EMBASE (n=643),  3) Cochrane(n=218),  4) AMED (n=32),  5) CNKI (n=61)  6) Cinii (n=507)  7) KoreaMed (n=8)  8) KMBASE (n=26)  9) OASIS (n=84)  10) NDSL (n=46)  11) KISS (n=40) | | |  | Additional records identified through other sources  (n=1) | | | |  | |
| --- | --- | --- | --- | --- | --- | --- | --- | --- | --- | --- | --- |
|  |  | ↓ | | |  | ↓ | | | |  | |
| **Screening** |  | Records after duplicates(n=1910) removed (n=364) | | | | | | | |  | |
|  |  |  | | ↓ | | |  | | |  | |
|  |  |  | Records screened  (n=1910) | | | | | → | Records excluded  (n=1847) | |  |
|  |  |  | | ↓ | | |  | | |  | |
| **Eligibility** |  |  | Full-text articles assessed for eligibility  (n=644) | | | | | → | Full-text articles excluded, with reasons  not intervention: 21  not RCT or proceeding: 38 | |  |
|  |  |  | | ↓ | | |  | | |  | |
| **Included** |  |  | Studies included in qualitative synthesis  (Q9 n=1)  (Q10 n=2)  (Q11 n=2) | | | | |  |  |  | |

**Eq. (C.5) Exercise (Q12)**

| **Identification** |  | Records identified through database searching  1) Medline (n=659),  2) EMBASE (n=570),  3) Cochrane(n=681),  4) AMED (n=131),  5) CNKI (n=0)  6) Cinii (n=107)  7) KoreaMed (n=7)  8) KMBASE (n=68)  9) OASIS (n=89)  10) NDSL (n=152)  11) KISS (n=92) | | |  | Additional records identified through other sources  (n=0) | | | |  | |
| --- | --- | --- | --- | --- | --- | --- | --- | --- | --- | --- | --- |
|  |  | ↓ | | |  | ↓ | | | |  | |
| **Screening** |  | Records after duplicates(n=1962) removed (n=595) | | | | | | | |  | |
|  |  |  | | ↓ | | |  | | |  | |
|  |  |  | Records screened  (n=1962) | | | | | → | Records excluded  (n=1812) | |  |
|  |  |  | | ↓ | | |  | | |  | |
| **Eligibility** |  |  | Full-text articles assessed for eligibility  (n=151) | | | | | → | Full-text articles excluded, with reasons  not P: 8  not I: 40  not C: 16  not O: 2  not RCT or proceeding: 74  duplicate: 6 | |  |
|  |  |  | | ↓ | | |  | | |  | |
| **Included** |  |  | Studies included in qualitative synthesis  (Q12 n=5) | | | | |  |  |  | |

**Eq. (C.6) Exercise (Q13)**

| **Identification** |  | Records identified through database searching  1) Medline (n=910),  2) EMBASE (n=1161),  3) Cochrane(n=520),  4) AMED (n=48),  5) CNKI (n=0)  6) Cinii (n=336)  7) KoreaMed (n=28)  8) KMBASE (n=68)  9) OASIS (n=163)  10) NDSL (n=199)  11) KISS (n=70) | | |  | Additional records identified through other sources  (n=0) | | | |  | |
| --- | --- | --- | --- | --- | --- | --- | --- | --- | --- | --- | --- |
|  |  | ↓ | | |  | ↓ | | | |  | |
| **Screening** |  | Records after duplicates(n=2547) removed (n=956) | | | | | | | |  | |
|  |  |  | | ↓ | | |  | | |  | |
|  |  |  | Records screened  (n=2547) | | | | | → | Records excluded  (n=1812) | |  |
|  |  |  | | ↓ | | |  | | |  | |
| **Eligibility** |  |  | Full-text articles assessed for eligibility  (n=199) | | | | | → | Full-text articles excluded, with reasons  not I: 49  not C: 30  not RCT or proceeding: 111  duplicate: 9 | |  |
|  |  |  | | ↓ | | |  | | |  | |
| **Included** |  |  | Studies included in qualitative synthesis  (Q13 n=0) | | | | |  |  |  | |

**Eq. (C.7) Mae-sun (Q14)**

| **Identification** |  | Records identified through database searching  1) Medline (n=17),  2) EMBASE (n=2),  3) Cochrane(n=11),  4) AMED (n=0),  5) CNKI (n=2)  6) Cinii (n=0)  7) KoreaMed (n=21)  8) KMBASE (n=0)  9) OASIS (n=84)  10) NDSL (n=0)  11) KISS (n=0) | | |  | Additional records identified through other sources  (n=0) | | | |  | |
| --- | --- | --- | --- | --- | --- | --- | --- | --- | --- | --- | --- |
|  |  | ↓ | | |  | ↓ | | | |  | |
| **Screening** |  | Records after duplicates(n=113) removed (n=24) | | | | | | | |  | |
|  |  |  | | ↓ | | |  | | |  | |
|  |  |  | Records screened  (n=113) | | | | | → | Records excluded  (n=106) | |  |
|  |  |  | | ↓ | | |  | | |  | |
| **Eligibility** |  |  | Full-text articles assessed for eligibility  (n=7) | | | | | → | Full-text articles excluded, with reasons  not P: 1  not I: 1  not C: 1  not RCT or proceeding: 3  duplicate: 1 | |  |
|  |  |  | | ↓ | | |  | | |  | |
| **Included** |  |  | Studies included in qualitative synthesis  (Q14 n=0) | | | | |  |  |  | |

**Eq. (C.8) Physical therapy (Q15,16)**

| **Identification** |  | Records identified through database searching  1) Medline (n=1316),  2) EMBASE (n=959),  3) Cochrane(n=750),  4) AMED (n=416),  5) CNKI (n=0)  6) Cinii (n=160)  7) KoreaMed (n=16)  8) KMBASE (n=56)  9) OASIS (n=110)  10) NDSL (n=118)  11) KISS (n=78) | | |  | Additional records identified through other sources  (n=1) | | | |  | |
| --- | --- | --- | --- | --- | --- | --- | --- | --- | --- | --- | --- |
|  |  | ↓ | | |  | ↓ | | | |  | |
| **Screening** |  | Records after duplicates(n=294) removed (n=1016) | | | | | | | |  | |
|  |  |  | | ↓ | | |  | | |  | |
|  |  |  | Records screened  (n= 2964) | | | | | → | Records excluded  (n=2632) | |  |
|  |  |  | | ↓ | | |  | | |  | |
| **Eligibility** |  |  | Full-text articles assessed for eligibility  (n=332) | | | | | → | Full-text articles excluded, with reasons | |  |
|  |  |  | | ↓ | | |  | | |  | |
| **Included** |  |  | Studies included in qualitative synthesis  (Q15 n=39)  (Q16 n=4) | | | | |  |  |  | |

**Forest plot & risk of bias table**

**1. Acupuncture (R1, R1-1, R2, R3)**

(1) R1. Pain VAS (Acupuncture vs sham or no-intervention controls)


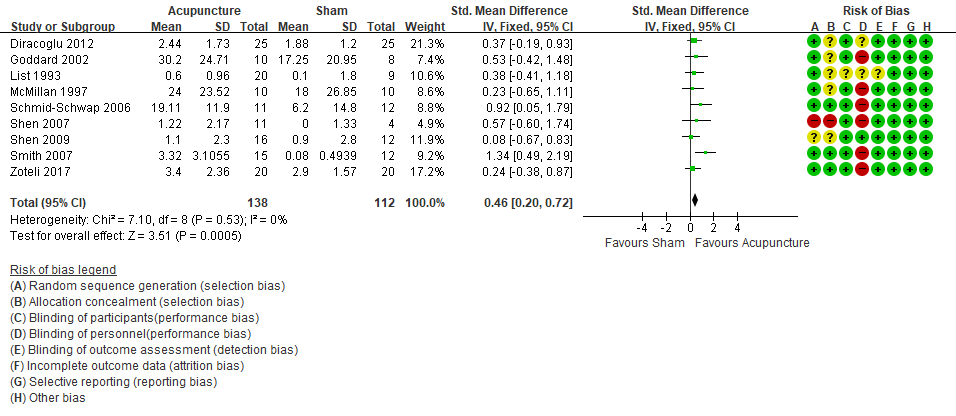


(2) R1. Maximum mouth opening (Acupuncture vs sham or no-intervention controls)


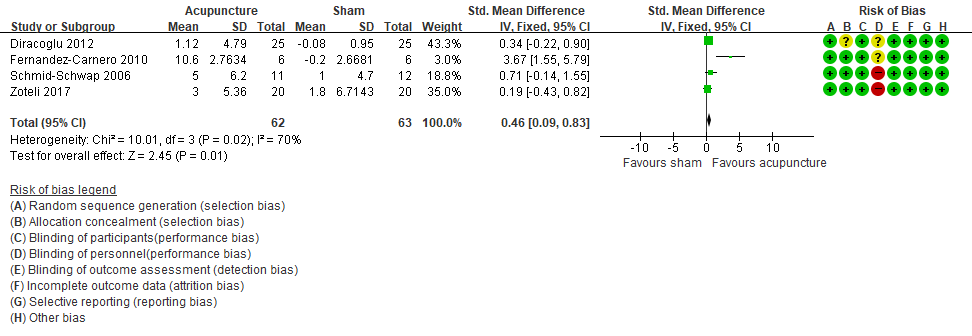


(3) R1. Effect rate (Acupuncture vs sham or no-intervention controls)


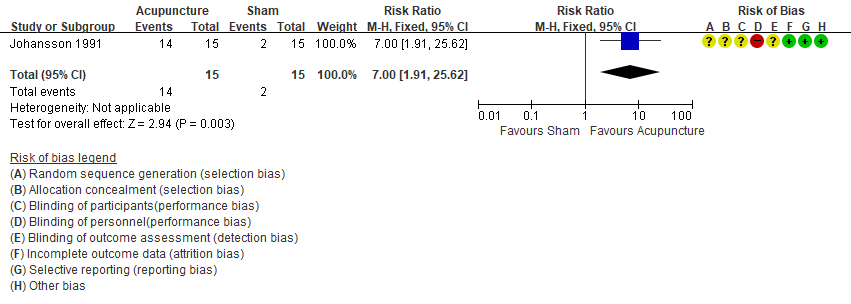


(4) R1-1. Pain (Acupuncture with remote acupuncture points vs neighboring acupuncture points vs combination of remote and neighboring acupuncture points)


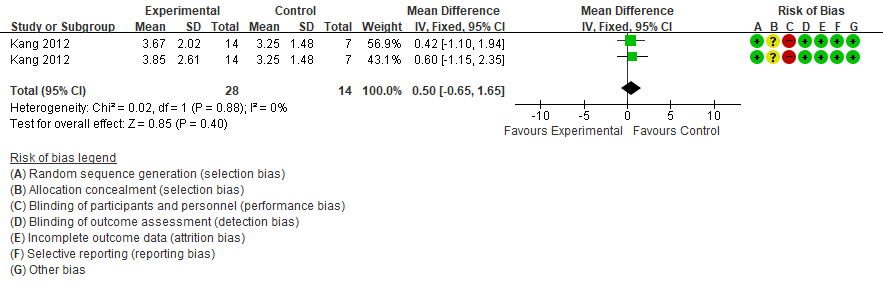


(5) R1-1. Function (Acupuncture with remote acupuncture points vs neighboring acupuncture points vs combination of remote and neighboring acupuncture points)


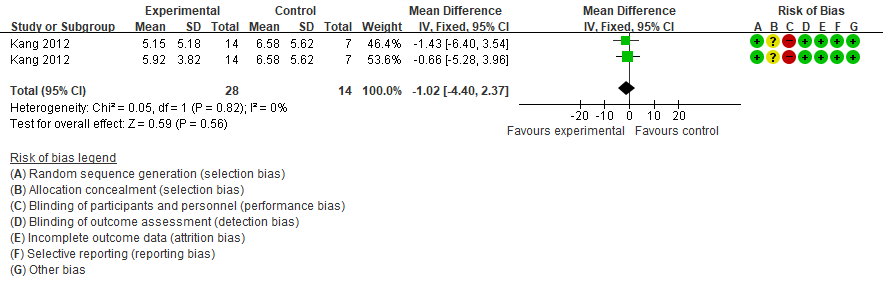
(6) R2. Effect rate (Acupuncture vs usual conservative treatment)


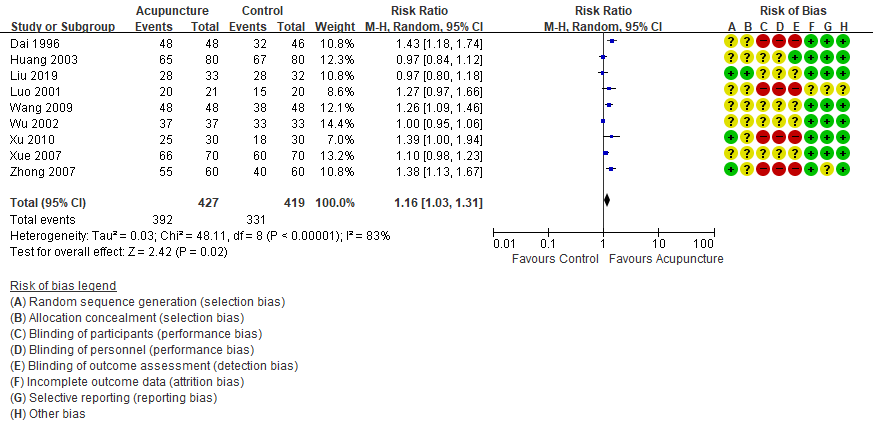


(7) R2. Pain (Acupuncture vs usual conservative treatment)


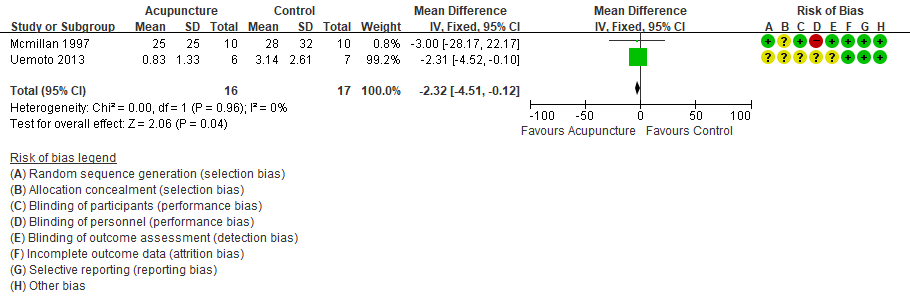


(8) R3. Effect rate (Acupuncture with usual conservative treatment vs usual conservative treatment)


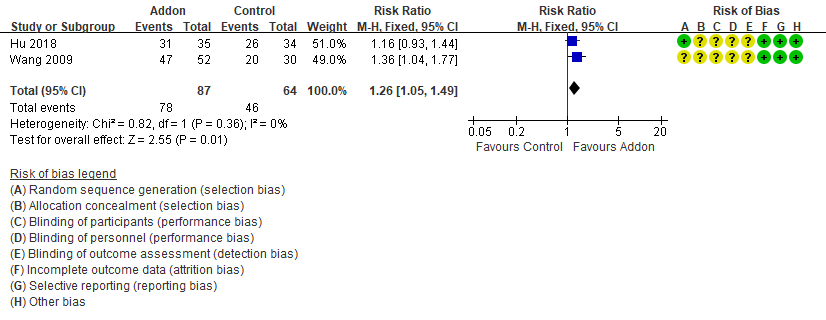


(9) R3. Pain VAS (Acupuncture with usual conservative treatment vs usual conservative treatment)


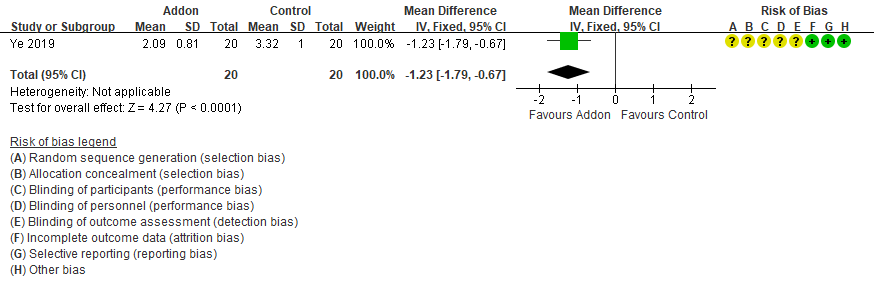


**2. Laser acupucnture (R4)**

(1) R4. Pain VAS (Laser acupucnture vs sham controls)


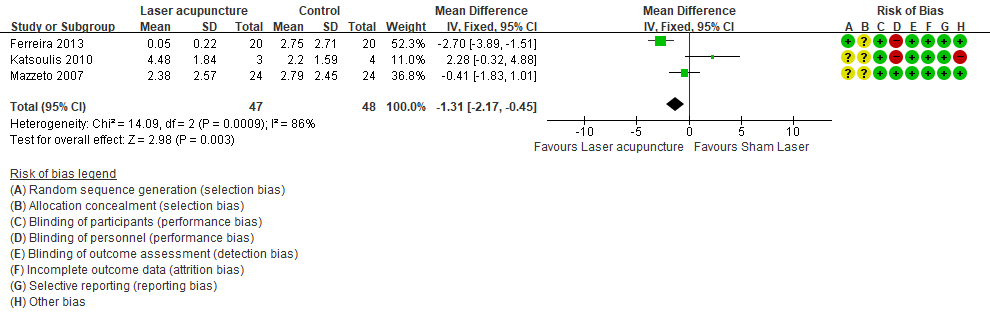


(2) R4. Maximum mouth opening (Laser acupucnture vs sham controls)


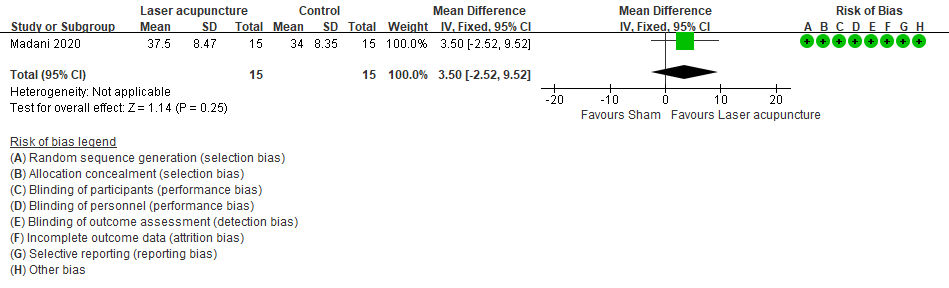


**3. Pharmacopuncture (R5)**

(1) R5. Pain VAS (Pharmacopuncture vs usual conservative treatment)


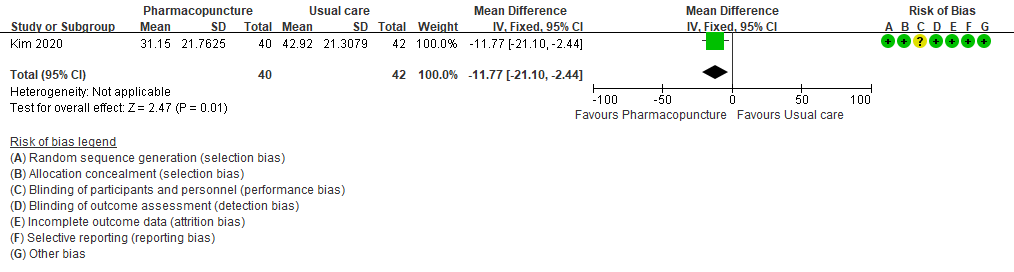


(2) R5. Pain NRS (Pharmacopuncture vs usual conservative treatment)


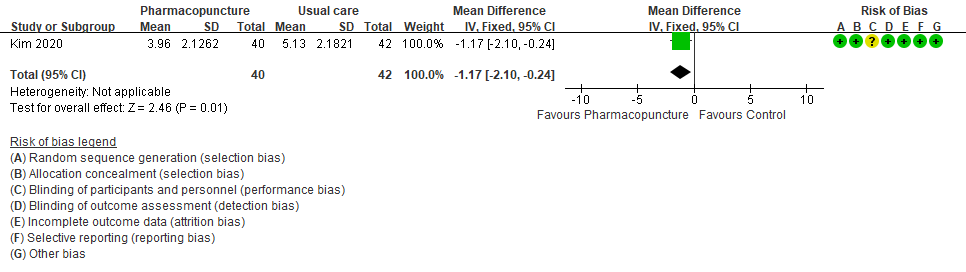
(3) R5. Discomfort NRS (Pharmacopuncture vs usual conservative treatment)


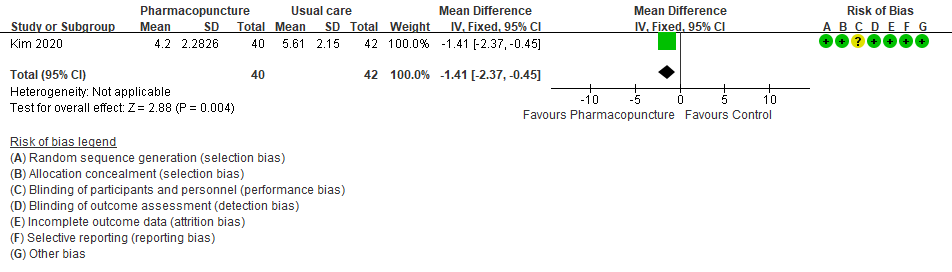


(4) R5. Maximum mouth opening (Pharmacopuncture vs usual conservative treatment)


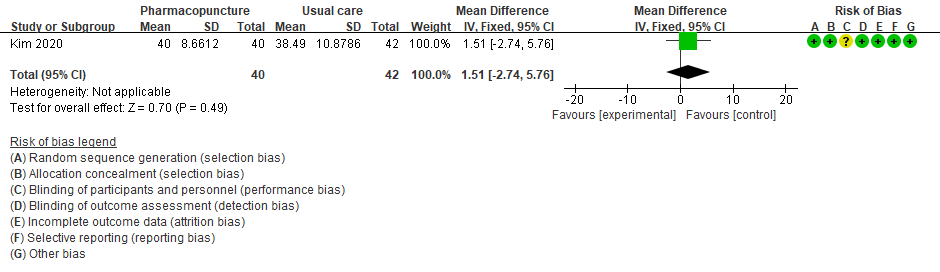


(5) R5. EQ-5D-5L (Pharmacopuncture vs usual conservative treatment)


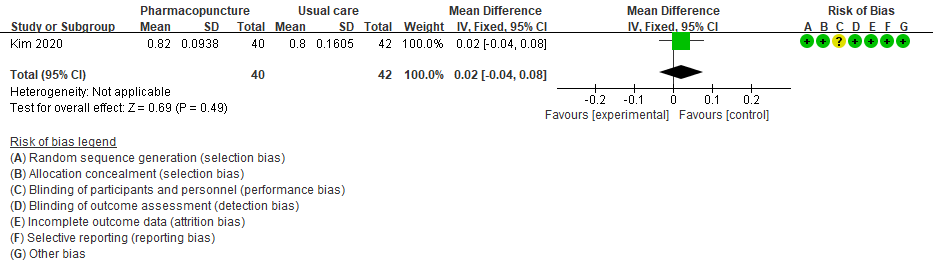


**4. Chuna manual therapy (R6, R7, R8)**

(1) R6. Pain VAS (Chuna manual therapy vs usual conservative treatment)


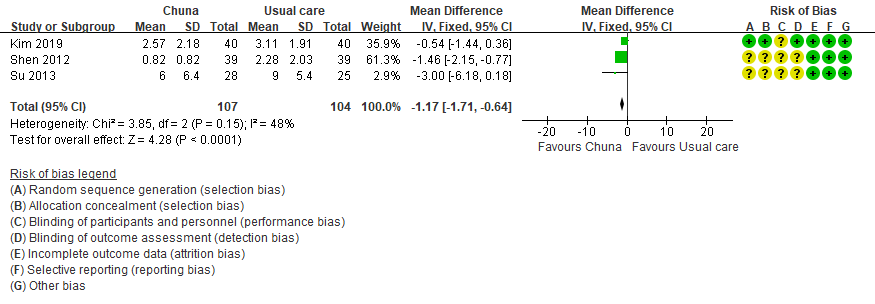
(2) R6. Maximum mouth opening (Chuna manual therapy vs usual conservative treatment)


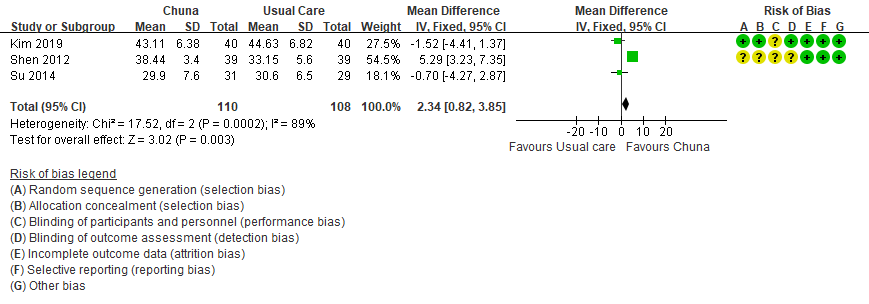


(3) R6. EQ-VAS change (Chuna manual therapy vs usual conservative treatment)


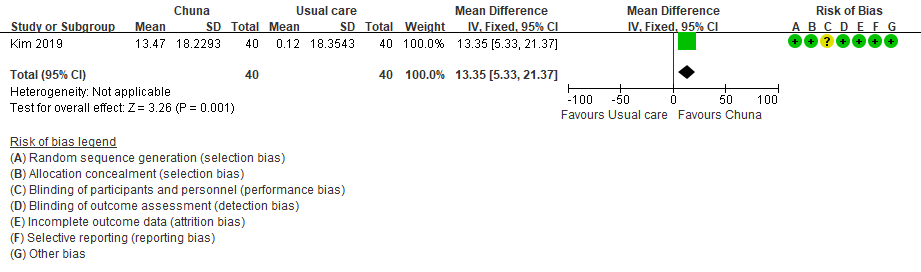


(4) R6. SF-12 PCS change (Chuna manual therapy vs usual conservative treatment)


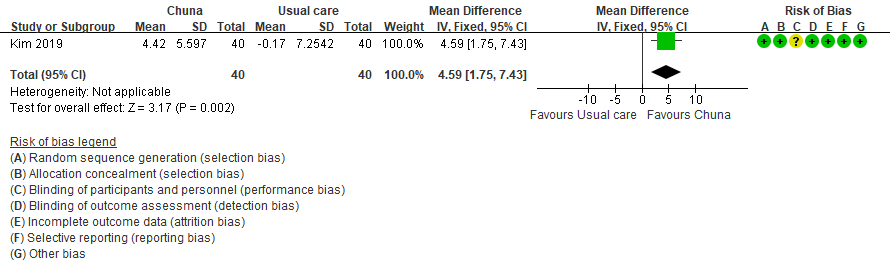
(5) R7. Effect rate (Chuna manual therapy with usual conservative treatment vs usual conservative treatment)


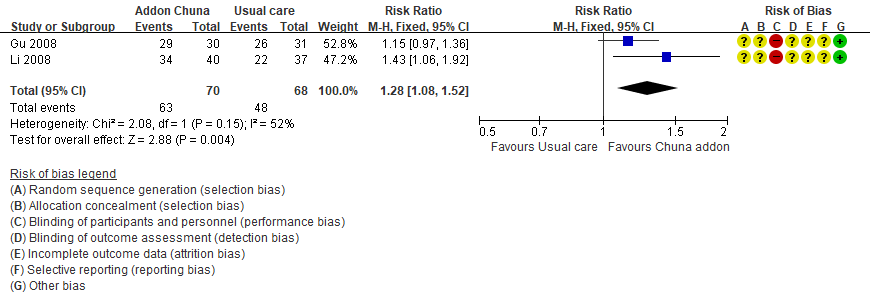


(6) R8. Effect rate (Chuna manual therapy with Korean meidicne treatment vs Korean meidicne treatment)


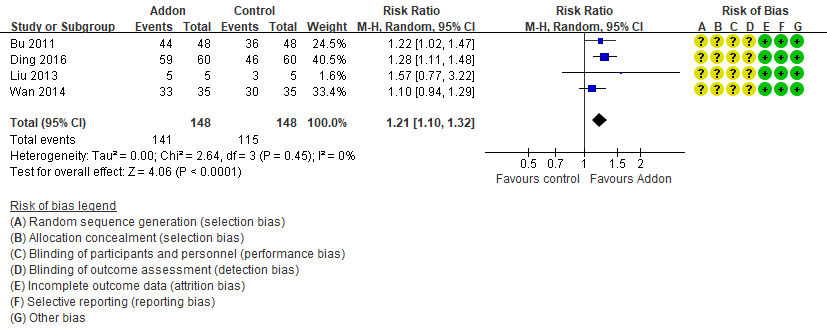


(7) R8. Pain VAS change (Chuna manual therapy with Korean meidicne treatment vs Korean meidicne treatment)


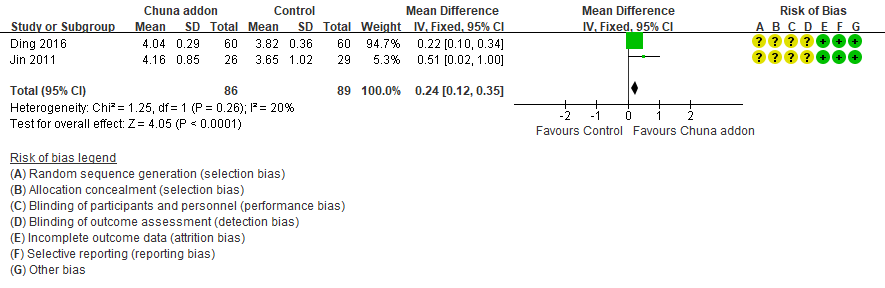


**5. Herbal medicine (R9, R10, R11)**

(1) R9. Effect rate (Herbal medicine vs usual conservative treatment)


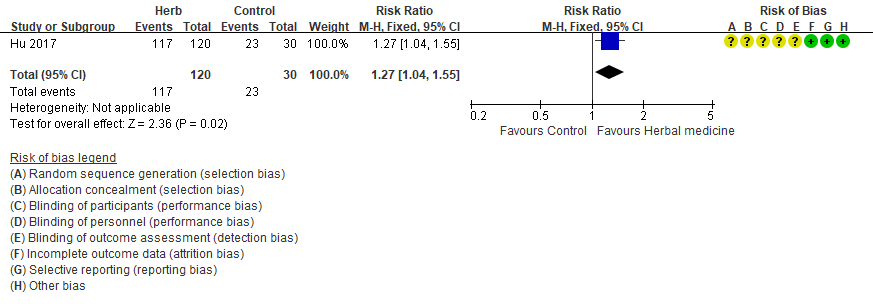


(2) R10. Effect rate (Herbal medicine with usual conservative treatment vs usual conservative treatment)


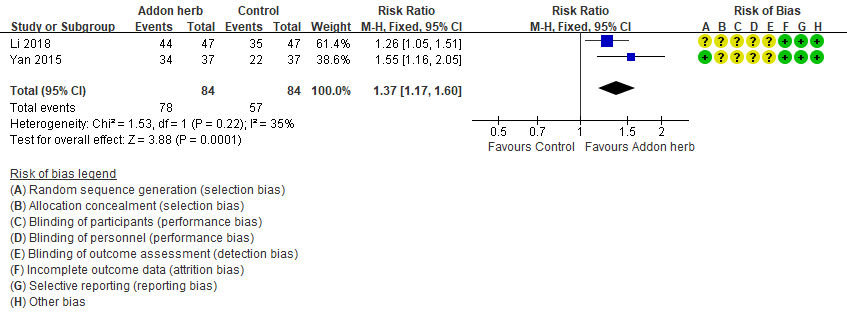


(3) R10. Pain VAS (Herbal medicine with usual conservative treatment vs usual conservative treatment)


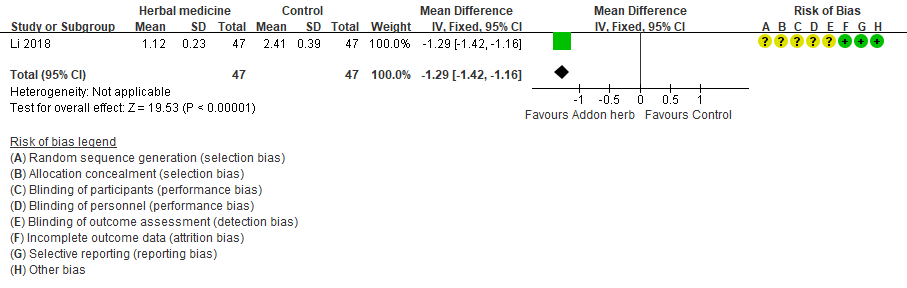
(4) R11. Effect rate (Herbal medicine with Korean meidicne treatment vs Korean meidicne treatment)


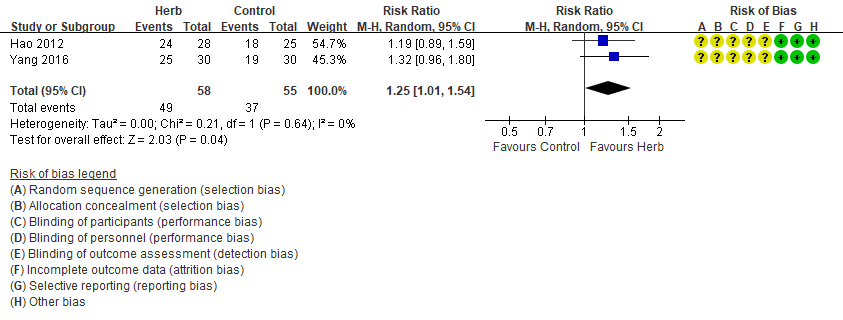


(5) R11. Pain VAS (Herbal medicine with Korean meidicne treatment vs Korean meidicne treatment)


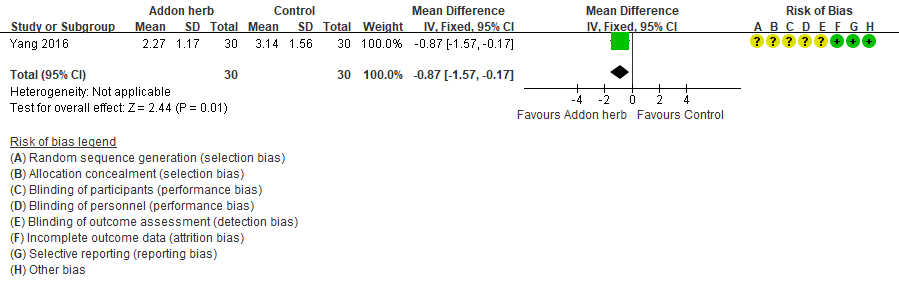


**6. Exercise therapy (R12)**

(1) R12. Pain VAS (Exercise vs non-active controls)


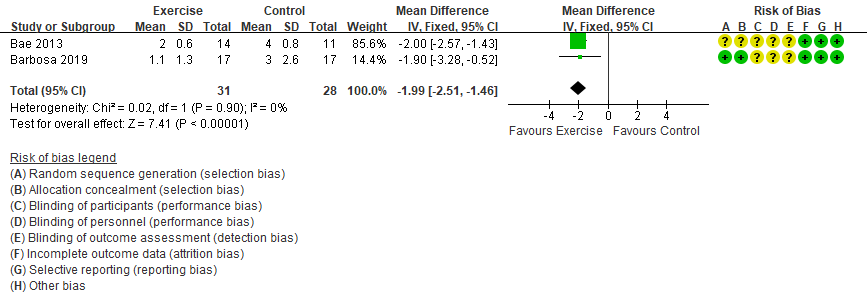


(2) R12. Maximum mouth opening (Exercise vs non-active controls)


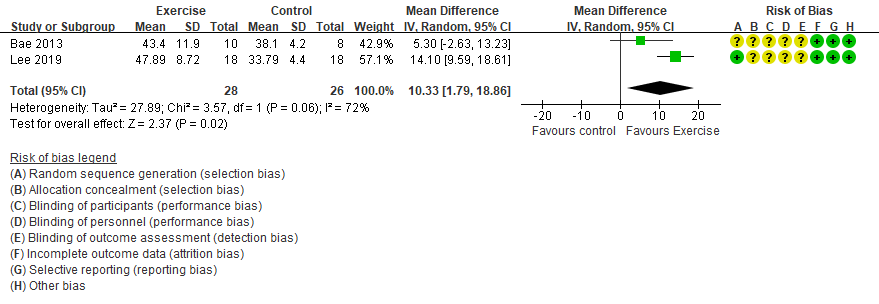


(3) R12. Effect rate (Exercise vs non-active controls)


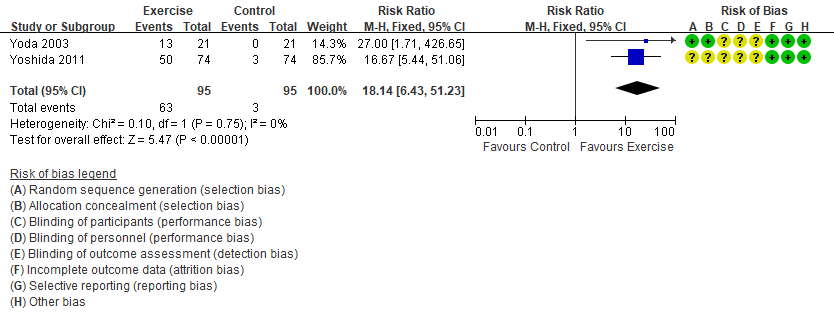


**7. Korean medicine physiotherapy (R15, R16)**

(1) R15. Pain VAS (Low-level laser therapy vs non-active controls)


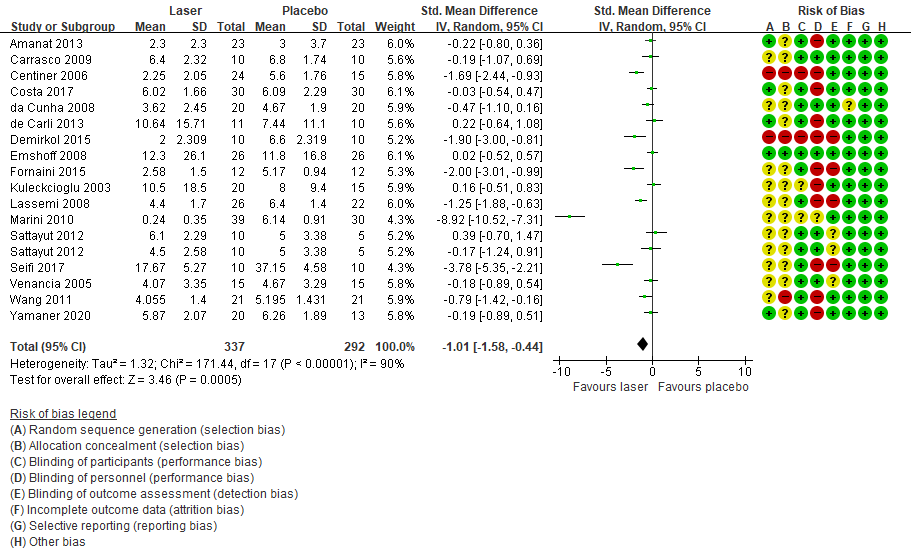


(2) R15. Pain VAS change (Low-level laser therapy vs non-active controls)


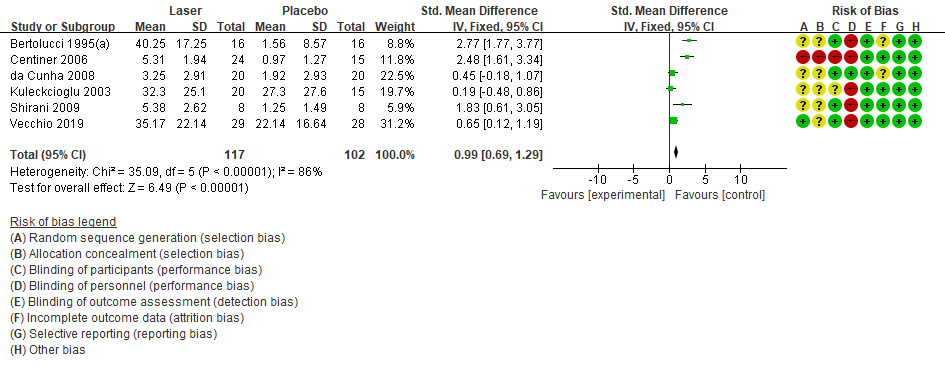


(3) R15. Maximum mouth opening (Low-level laser therapy vs non-active controls)


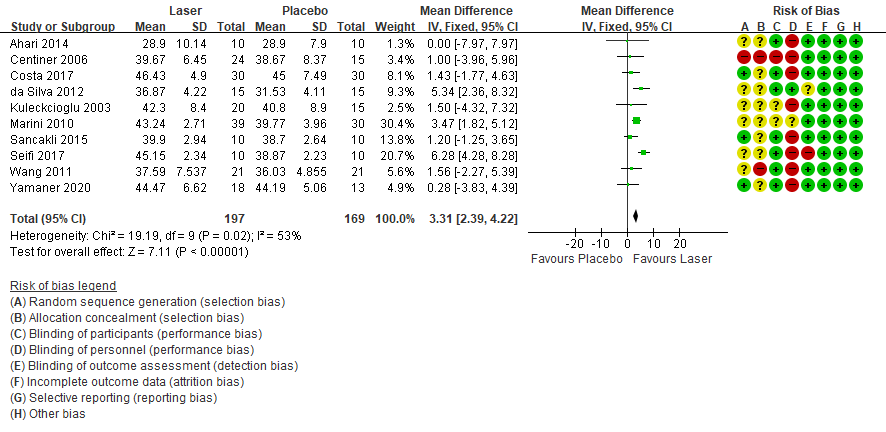


(4) R15. Maximum mouth opening change (Low-level laser therapy vs non-active controls)


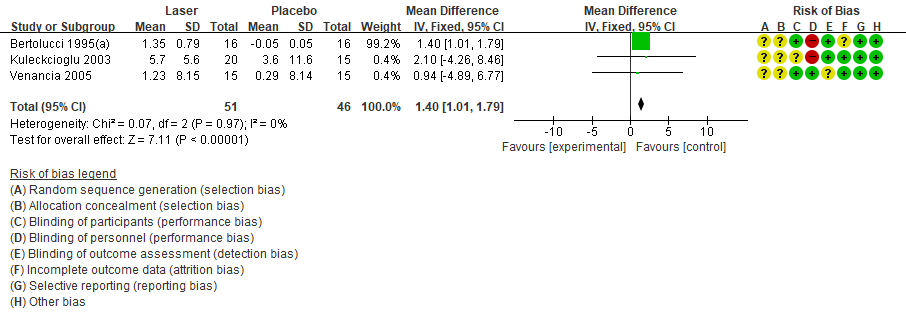


(5) R15. Pain VAS (TENS vs non-active controls)


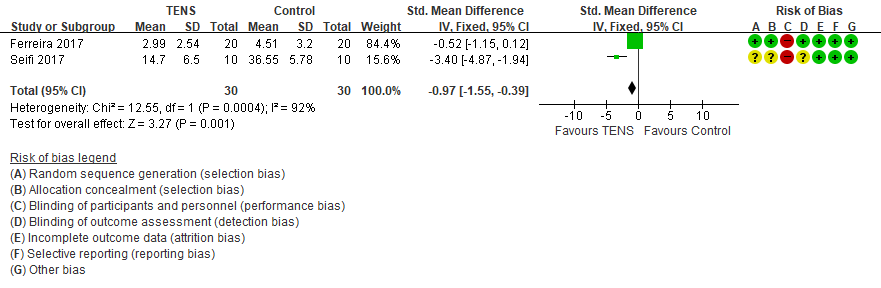


(6) R15. maximum mouth opening (TENS vs non-active controls)


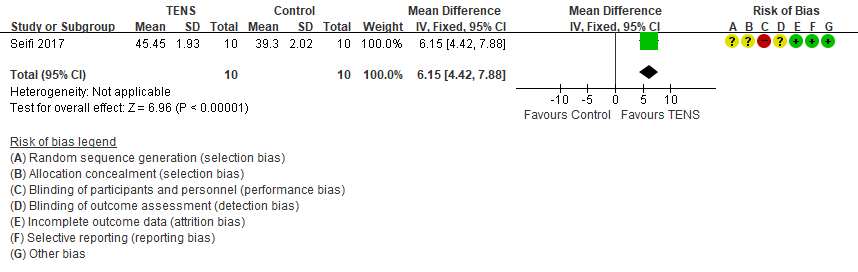


(7) R16. Pain VAS change (Korean medicine physiotherapy with usual conservative treatment vs usual conservative treatment)


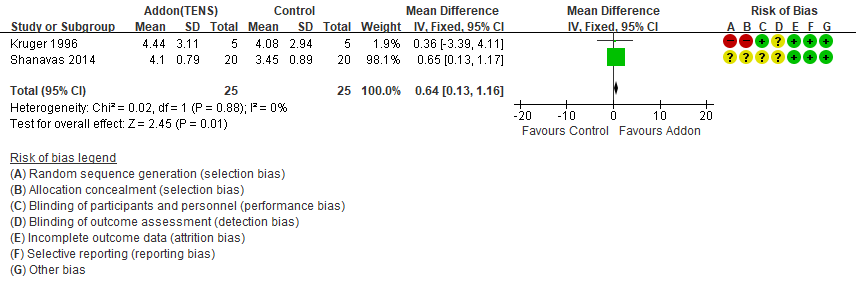


**8. Combination of Korean medicine treatments (R17)**

(1) R17. Worst pain (Combination of Korean medicine treatments vs usual conservative treatment)
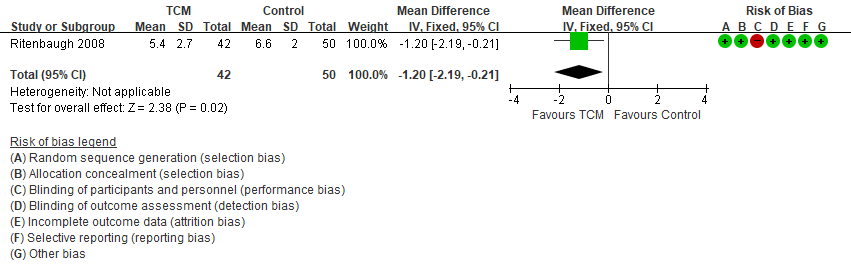


(2) R17. Average pain (Combination of Korean medicine treatments vs usual conservative treatment)


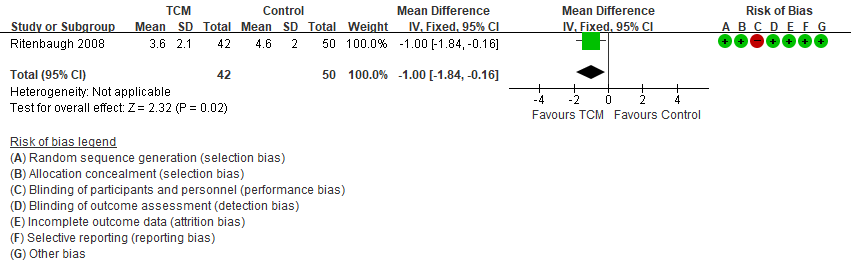

Supplement: Supplementary file 1 [file healthcare-11-02364-s001.zip › healthcare-2547795-supplementary/Supplementary Materials S1.docx]
