# Supplementary material for: Korean Medicine Clinical Practice Guideline Update for Temporomandibular Disorders: An Evidence-Based Approach
Source: Healthcare (Basel). 2023 Aug 21;11(16):2364. doi: 10.3390/healthcare11162364 (PMC10454242; doi:10.3390/healthcare11162364)
Supplement: Supplementary file 1 [file healthcare-11-02364-s001.zip › healthcare-2547795-supplementary/Supplementary Materials S2.pdf]

Evidence Table

Eq. (E.1) [Q1] Acupuncture Group VS Inactive Control Group

No.	Study ID	Study design	Country (period)	Intervention (n)	Comparison (n)	Disease of participants (Age : Mean±S.D)	Number of Participants	Acupoints	Session or Treatment period	Relevant outcomes	Adverse events
1	Diracoglu et al. (2012)	RCT	Turkey (NR)	Dry needling	Sham dry needling	Temporomandibular myofascial pain (IG: 33.00±12.70 CG: 35.88±9.60)	IG: 26 CG: 26	TrPs in masseter and temporalis muscles	3 times with 7-day intervals	1) PPT 2) VAS 3) unassisted jaw opening	NR
2	Goddard et al. (2002)	RCT	USA (NR)	Acupuncture	Sham acupuncture	Myofascial pain (IG: 35.49±10.63 CG: 34.53±6.78)	IG: 10 CG: 8	LI4, ST6	One time	VAS	NR
3	Itoh et al. (2012)	RCT	Japan (NR)	TrP Acupuncture	Sham acupuncture	Chronic TMJ myofascial pain (IG: 21.7±2.1 CG: 21.4±1.4)	IG: 8 CG: 8	Myofascial TrPs	Total 5 sessions, Once a week	1) VAS 2) MMO	NR

No.	Study ID	Study design	Country (period)	Intervention (n)	Comparison (n)	Disease of participants (Age : Mean±S.D)	Number of Participants	Acupoints	Session or Treatment period	Relevant outcomes	Adverse events
4	Schmid-Schwap (2006)	RCT	Austria (11/2001-06/2003)	Acupuncture	Sham acupuncture	females with TMJ pain and tenderness on pressure of the craniomandibular musculature (IG: 35±14 CG: 40±14)	IG: 11 CG: 12	Intraoral: Maxilla retromolar, Mandible retromolar, Maxilla - vestibulum and Mandible - vestibulum; extraoral: large intestine 4, small intestine 2 and 3 (hand), ear and sternum.	One time	1) VAS 2) MMO	None
5	Smith (2007)	RCT	UK (06-07/2003)	Acupuncture	Sham acupuncture	TMJ MP for at least 6 months (IG: 38.3±13.39 CG: 43.2±4.04)	IG: 15 CG: 12	ST7	6 sessions for 3 weeks. all outcome measures 3 days and then 7 days following the final acupuncture session	1) VAS Functional impairment Pain intensity Pain distribution	NR

No.	Study ID	Study design	Country (period)	Intervention (n)	Comparison (n)	Disease of participants (Age : Mean±S.D)	Number of Participants	Acupoints	Session or Treatment period	Relevant outcomes	Adverse events
6	Shen et al. (2007)	RCT	USA (NR)	Acupuncture	Sham acupuncture	Chronic MPS of masticatory muscles (IG: 45.2±12.3 CG: 41.8±14.9)	IG: 9<br>CG: 6	LI4	One time	1) NRS<br>Facial pain<br>Neck pain<br>Headache<br>2) VAS<br>Mechanical pressure pain	NR
7	Shen et al. (2009)	RCT	USA (NR)	Acupuncture	Sham acupuncture	MPS of jaw muscles (IG: 36.94±13.82 CG: 44.83±11.61)	IG: 16<br>CG: 12	LI4	One time	1) NRS<br>jaw pain<br>jaw/face tightness<br>headache<br>neck pain<br>2) VAS<br>pain tolerance of the masseter muscle	NR

No.	Study ID	Study design	Country (period)	Intervention (n)	Comparison (n)	Disease of participants (Age : Mean±S.D)	Number of Participants	Acupoints	Session or Treatment period	Relevant outcomes	Adverse events
8	Simma (2009)	RCT	Austria (02/2002-07/2003)	Acupuncture	placebo laser	dysfunction and pain in TMJ(NR)	IG: 11 CG: 12	Upper jaw retromolar Lower jaw retromolar Upper jaw vestibulum Lower jaw vestibulum Large intestine 4 Small intestine 3, 2 Auricle Sternum Adler Langer points	One time	1) Pain rating by palpation of 14 muscles 2) VAS	NR
9	Shen et al. (2009)	RCT	USA (NR)	Acupuncture	Sham acupuncture	MPS of jaw muscles (IG: 37.33±12.97 CG: 44.5±13.7)	IG: 6 CG: 6	LI4	One time	fMRI	NR

No.	Study ID	Study design	Country (period)	Intervention (n)	Comparison (n)	Disease of participants (Age : Mean±S.D)	Number of Participants	Acupoints	Session or Treatment period	Relevant outcomes	Adverse events
10	Zotelli (2017)	RCT	BRAZIL(07/2015-06/2016)	Acupuncture	Sham acupuncture	temporomandibular disorders (TMD) (IG: 38±8.7 CG: 35.1±8.5)	IG: 23<br>CG: 20	ST6, ST7, SI18, GV20, GB20, BL10, LI4	Once a week, 4 sessions	1) MMO<br>Unassisted painless mouth opening<br>Unassisted mouth opening<br>Assisted mouth opening<br>2) VAS	NR
11	Lopez-Martos (2018)	RCT	SPAIN (06/2015-06.2016)	1) PNE (percutaneous needle electrolysis)<br><br>2) DDN (deep dry needling)	Sham acupuncture	myogenic pain in the temporo-mandibular area of at least 6 months (IG1: 38.5 (18-57) IG2: 36 (19-58) CG: 42 (25-62))	IG1: 20<br>IG2: 20<br>CG: 20	IG1 : transcutaneous puncture in the LPM<br><br>IG2 : TPs<br><br>CG : LPM	One session per a week for 3 weeks	1) VAS<br>PNE vs DDN<br>PNE vs SNP<br>DDN vs SNP	NR

No.	Study ID	Study design	Country (period)	Intervention (n)	Comparison (n)	Disease of participants (Age : Mean±S.D)	Number of Participants	Acupoints	Session or Treatment period	Relevant outcomes	Adverse events
11	Fernández-Carnero (2010)	RCT	SPAIN (01-07/2008)	Dry needling	Sham dry needling	myofascial pain according to the Research Diagnostic Criteria for TMD (25±6)	IG: 6<br>CG: 6	TrP on the masseter muscle (most painful point on the masseter muscle)	2 sessions at least 7 days apart (assigned in a random fashion at each visit)	1) PPT masseter muscle mandibular condyle<br>2) MMO	NR
12	Johansson (2006)	RCT	SWEDEN (NR)	Acupuncture	CG1: Splint<br>CG2: No treatment	CMD (NR)	IG: 15<br>CG1: 15<br>CG2: 15	Three to seven needles were used locally and LI4	6 sessions for IG	1) VAS<br>IG vs CG1<br>IG vs CG2<br>CG1 vs CG2<br>2) SDS<br>IG vs CG1<br>IG vs CG2<br>CG1 vs CG2<br>3) ER<br>IG vs CG1<br>IG vs CG2<br>CG1 vs CG2<br>4) CDS<br>IG vs CG1<br>IG vs CG2<br>CG1 vs CG2	NR

No.	Study ID	Study design	Country (period)	Intervention (n)	Comparison (n)	Disease of participants (Age : Mean±S.D)	Number of Participants	Acupoints	Session or Treatment period	Relevant outcomes	Adverse events
13	McMillan (1997)	RCT	UK (NR)	IG1: Procain + simulated dry needling IG2: Dry needling + simulated local anesthetic	CG: simulated dry needling + simulated local anesthetic	TMD with MPS (23–53)	IG1: 10 IG2: 10 CG: 10	active TP in the masseter	3 occasions 1 week apart	1) PPT masseter muscle temporalis muscle 2) VAS Intensity Unpleasantness	NR
14	List (1993)	RCT	SWEDEN (NR)	IG1: Acupuncture IG2: Splint	CG: No treatment	cramomandibular disorders and a history of pain of at least 6 months (22–69)	IG1: 20 IG2: 20 CG: 15	Ex2, ST7, ST6, GB20, LI4, ST36	treatment period lasted between 6 and 8 weeks, 1 session was given at 1–week intervals	1) PPT 2) CDS 2) VAS	NR

Eq. (E.2) [Q2] Acupuncture Group VS usual Conservative Treatment Group

No.	Study ID	Study design	Country (period)	Intervention (n)	Comparison (n)	Disease of participants (Age : Mean±S.D)	Number of Participants	Acupoints	Session or Treatment period	Relevant outcomes	Adverse events

No.	Study ID	Study design	Country (period)	Intervention (n)	Comparison (n)	Disease of participants (Age : Mean±S.D)	Number of Participants	Acupoints	Session or Treatment period	Relevant outcomes	Adverse events
1	McMillan (1997)	RCT	UK (NR)	IG1: Procain + simulated dry needling IG2: Dry needling + simulated local anesthetic	CG: simulated dry needling + simulated local anesthetic	TMD with MPS (23-53)	IG1: 10 IG2: 10 CG: 10	active TP in the masseter	3 occasions 1 week apart	1) PPT masseter muscle temporalis muscle 2) VAS Intensity Unpleasantness	NR
2	Gonzalez-Perez et al. (2015)	RCT	Spain (NR)	deep dry needling (DDN) of trigger points (TPs)	methocarbamol/paracetamol medication.	temporo-mandibular myofascial pain located in the lateral pterygoid muscle (IG: 34.3 ± 13.8, CG: 35.5 ± 11.2)	IG: 24 CG: 24	lateral pterygoid muscle (LPM)	3 times with 7-day intervals	IG: once per week for 3 weeks CG: dose of two tablets every six hours for three weeks	NR
3	Dai et al. (1996)	RCT	China (NR)	Warm needling	TDP	Temporomandibular joint syndrome (NR)	IG: 48 CG: 46	Ashipoint, ST6, SI19 (affected),, LI4(bilateral)	10 sessions, 간격 3-5일	Effective rate	

No.	Study ID	Study design	Country (period)	Intervention (n)	Comparison (n)	Disease of participants (Age : Mean±S.D)	Number of Participants	Acupoints	Session or Treatment period	Relevant outcomes	Adverse events
4	Luo et al. (2001)	RCT	China (NR)	Acupuncture	very high frequency	Temporomandibular joint syndrome (NR)	IG: 21 CG: 20	first group: GB2, ST7, GB20 second group: SI19, TE17, LI4	10 sessions, 5 days intervals	Effective rate	
5	Xu et al. (2010)	RCT	China (2008/07–2009/06)	Surrounding Electroacupuncture	local ultrashort wave	temporomandibular joint disturbance syndrome (IG: 35±5, CG: 34±4)	IG: 30 CG: 30	Ashipoint	daily, 10 sessions	McGill pain questionnaire (VAS, PRI, PPI)	
6	Zhong et al. (2007)	RCT	China (2005/12–2007/01)	Warm needling	ultrasound	temporomandibular joint disturbance syndrome (temporomandibular disorder) (IG: 35±5, CG: 34±4)	IG: 60 CG: 60	ST6, ST7	daily, 10 sessions	efficacy rate	

No.	Study ID	Study design	Country (period)	Intervention (n)	Comparison (n)	Disease of participants (Age : Mean±S.D)	Number of Participants	Acupoints	Session or Treatment period	Relevant outcomes	Adverse events
7	Huang et al. (2003)	RCT	China (NR)	IG : Acupuncture+ diclofenac magma+ TDP	CG1 : Computer pulse massage CG2 : Acupuncture	TMJD muscle group dysfunction (Class I, 129 cases) joint structural disorders (Class II, 94 cases) less organic damage (Class III, 17 cases)	IG: 80, CG1: 80 CG2: 80	ST7, ST6, LI4	NR, 10days	Effective rate	
8	Wu et al. (2002)	RCT	China (NR)	Warm needling	Diclofenac sodium	Temporomandibular Joint Disturbance Syndrome	IG: 37 CG: 33	ST7	daily/7days	Effective rate	
9	da silve (2012)	RCT	Brazil (NR)	deep dry needling	needling + injection lidocaine hydrochloride 0.5%.	TMD index, RDC / TMD	16	myofascial pain trigger points	One time	1) PPT 2) VAS	
10	Liu (2019)	RCT	China (2015/09-2017/09)	Warm needling	diclofenac sodium sustained-release capsules	Temporomandibular Joint Disturbance Syndrome (IG: 48±0, CG: 49±1)	IG: 33 CG: 32	Ashipoint, LI4	3 times a week / 4 weeks.	1)VAS 2)Friction index	

No.	Study ID	Study design	Country (period)	Intervention (n)	Comparison (n)	Disease of participants (Age : Mean±S.D)	Number of Participants	Acupoints	Session or Treatment period	Relevant outcomes	Adverse events
11	Dalewski et al. (2019)	RCT	Poland (2016/07~2017/12)	Dry needling/ NSAIDs	Occlusal appliance	Temporomandibular disorder−Chronic orofacial pain (IG: 31.3 IG: 31.2 CG: 28.7)	IG: 30 IG: 30 CG: 30	Myofascial TrPs	3 times with 7−day intervals	1) VAS 2) SPAQ(Sleep and Pain Activity Questionnaire)	NR
12	Aksu et al. (2019)	RCT	Turkey (2013/03~2013/09)	Dry needling/ TrP inj.	Exercise, PT	Temporomandibular myofascial pain (IG+CG: 39.4±14.9)	IG: 20 IG: 22 CG: 21	TrP in both masseter and lateral pterygoid muscles	3 times with a weekly intervals	VAS	NR
13	Uemoto et al. (2013)	RCT	Brazil(NR)	Laser/ Dry needling in myofascial TrP	Placebo treatment TrP	Temporomandibular joint disorders(TMJD) (NR)	IG: 7 IG: 7 CG: 7	TrPs in right masseter muscle	4 times with intervals ranging between 48 and 72h	VAS	NR

No.	Study ID	Study design	Country (period)	Intervention (n)	Comparison (n)	Disease of participants (Age : Mean±S.D)	Number of Participants	Acupoints	Session or Treatment period	Relevant outcomes	Adverse events
14	Eroglu et al. (2013)	RCT	Turkey (2009/04~2009/07)	Dry needling/lidocaine inj./Oral flurbiprofen 200mg/day		Myofascial pain syndrome (IG: 33.75±8.10, IG: 32.85±9.06, IG: 34.55±8.30)	IG: 20<br>IG: 20<br>IG: 20	all active trigger points	3times (1st day, 3rd day, 14th day)	1) VAS<br>2) Degree of tenderness at the trigger points by algometry<br>3) Active joint ROM of the neck and shoulders by goniometry<br>4) Quality of life by Nottingham Health Profile	NR
15	Xue et al. (2007)	RCT	China(2004/06~2006/06)	Warming needle moxibustion plus exercise	Simple filiform needle needling/local blocking therapy	Temporomandibular joint dysfunction syndrome (NR)	IG: 70<br>IG: 70<br>IG: 70	LI4, ST7, TE17	10times	邱蔚六, standard for orofacial surgery(total outcome according to symptoms, range of movement, etc.)	

No.	Study ID	Study design	Country (period)	Intervention (n)	Comparison (n)	Disease of participants (Age : Mean±S.D)	Number of Participants	Acupoints	Session or Treatment period	Relevant outcomes	Adverse events
16	Wang et al. (2009)	RCT	China(2006/06~2007/08)	Electroacupuncture	partial closure method	Temporomandibular disorders (IG: 40.7 CG: 38.5)	IG: 48 CG: 48	ST7, ST6, LI4, Ashipoint	7times	邱蔚六, standard for orofacial surgery(total outcome according to symptoms, range of movement, etc.)	

Eq. (E.3) [Q2-1] Distal Acupoints Group VS Local Acupoints Group VS Concurrent Treatment of Distal and Local Acupoints Group

No.	Study ID	Study design	Country (period)	Intervention (n)	Comparison (n)	Disease of participants (Age : Mean±S.D)	Number of Participants	Acupoints	Session or Treatment period	Relevant outcomes	Adverse events
1	Kang (2012)	RCT	Korea	IG : Adjacent point treatment group (Trt)	CG1 : distant-point acupuncture (Con1), CG2 : combined acupuncture of an adjacent and a distant point (Con2)	unilateral or bilateral TMD diagnosed following the Research Diagnostic Criteria and were required to have an AxisI, Group I diagnosis	IG : 12<br>CG1 : 12<br>CG2 : 14	TE17, GB20, ST7, ST6, SI19, EX21	six acupuncture sessions (twice a week for 3weeks)	1) VAS<br>IG vs CG1 vs CG2<br>2) Muscle and TMJ palpation index<br>IG vs CG1 vs CG2	IG : dental pain D/O<br>CG1 : gum and mouth pain D/O

Eq. (E.4) [Q3] The Concurrent Treatment Group of Acupuncture and usual Conservative Treatment VS usual Conservative Treatment Group

No.	Study ID	Study design	Country (period)	Intervention (n)	Comparison (n)	Disease of participants (Age : Mean±S.D)	Number of Participants	Acupoints	Session or Treatment period	Relevant outcomes	Adverse events
1	Dalewski et al. (2019)	RCT	Poland (2016/07~2017/12)	Dry needling/ NSAIDs	Occlusal appliance	Temporomandibular disorder-Chronic orofacial pain (IG: 31.3 IG: 31.2 CG: 28.7)	IG: 30 IG: 30 CG: 30	Myofascial TrPs	3 times with 7-day intervals	1) VAS 2) SPAQ(Sleep and Pain Activity Questionnaire)	NR
2	Aksu et al. (2019)	RCT	Turkey (2013/03~2013/09)	Dry needling/ TrP inj.	Exercise, PT	Temporomandibular myofascial pain (IG+CG: 39.4±14.9)	IG: 20 IG: 22 CG: 21	TrP in both masseter and lateral pterygoid muscles	3 times with a weekly intervals	VAS	NR
3	Wang et al. (2009)	RCT	China (2013/05~)	Acupuncture combined with magnetic therapy	only magnetic therapy	Temple-jaw joint dysfunction (IG: 30.48±11.2 CG: 31.5±12.1)	IG: 52 CG: 30	ST7, ST6, LI4	10 times everyday	邱蔚六, standard for orofacial surgery(total outcome according to symptoms, range of movement, etc.)	NR

No.	Study ID	Study design	Country (period)	Intervention (n)	Comparison (n)	Disease of participants (Age : Mean±S.D)	Number of Participants	Acupoints	Session or Treatment period	Relevant outcomes	Adverse events
4	Ye et al. (2019)	RCT	China (2018/01~2018/10)	Very High Frequency and Electroacupuncture	Very High Frequency	TMD (IG: 44.05±12.40 CG: 42.05±10.88)	IG: 20 CG: 20	SI19, ST7, ST6, LI4, ST36 + 先天不足, 肝腎虛者 KI3, BL18 配合 (實證은 瀉法, 虛證은 補法)	10 times in 2 weeks (5 times a week)	1) VAS 2) OR(range of motion)	NR
5	Hu et al. (2018)	RCT	China(2013/09−2016/03)	Very High Frequency and Electroacupuncture	Very High Frequency	TMD (NR)	IG: 35 CG: 34	SI19, TE17, ST7, ST6, Ashipoint, LI4, GB34, LR3, ST36	7 times everyday	邱蔚六, standard for orofacial surgery(total outcome according to symptoms, range of movement, etc.)	NR

Eq. (E.5) [Q4] Laser Therapy Group VS Inactive Control Group

No.	Study ID	Study design	Country (period)	Intervention (n)	Comparison (n)	Disease of participants (Age : Mean±S.D)	Number of Participants	Acupoints	Session or Treatment period	Relevant outcomes
1	Ferreira et al. (2013)	RCT	Brazil (NR)	Laser acupuncture + NMOS	Placebo laser + NMOS	Chronic myofascial pain and arthralgia (34.17±8.83)	IG: 20<br>CG: 20	ST6, I19, B20, LI4, LR3, TE3, GB34, EX-HN3	Once a week for 3 months	VAS
2	Katsoulis et al. (2010)	RCT	Switzerland(NR)	Laser needle acupuncture	Placebo laser needle acupuncture	tendomyopathy of the masticatory musculature (33, NR)	IG(open): 4<br>IG(blinded): 3<br>CG: 4	ST6, SI18, SI 3, LI 4	two 15 min sessions per week for 3 weeks	VAS<br>Verbal scale

Eq. (E.6) [Q5] Pharmacopuncture Group VS usual Conservative Treatment Group

No.	Study ID	Study design	Country (period)	Intervention (n)	Comparison (n)	Disease of participants (Age : Mean±S.D)	Number of Participants	Acupoints	Session or Treatment period	Relevant outcomes	Adverse events
1	Kim et al. (2020)	RCT	Korea (NR)	Hominis placental pharmacopuncture (n=41)	Physical therapy (n=41)	Chronic TMD (NR)	IG: 41<br>CG: 41	SI19, GB20, GB21, TE17, ST7, ST6, LI18, EX-HN5	10 times (twice in a week during 5 weeks)	1) VAS<br>2) NRS	NR

Eq. (E.7) [Q6] Chuna Manipulation Group VS usual Conservative Treatment Group

No.	Study ID	Study design	Intervention (n)	Comparison (n)	Duration	F/U	Outcome measurements	Results	Adverse events
1	Kim et al. (2019)	RCT (protocol)	Chuna manual therapy	Usual care (UC) [thermotherapy, ultrasound therapy, TENS, ICT, etc.]	8 sessions for 4 weeks	1st, 3rd, 5th, and 7th treatment visits (week 1-4) and 5 weeks, 3 months, and 6 months	VAS, NRS, ROM, BDI, JFLS, PGIC, SF-12, EQ5D-5L	-	Total 6 events (추나군: 3, Usual care:3) 추나군: 두통, 이명, 구강점막 부종 Usual care: 귀통증, 목통증, 턱관절 통증 심화호소
2	Chen et al. (2012)	RCT	TMT (acupoint massage)	Western medication (Meloxicam Tablets and Composite Chlorzozazone Tablets)	1 session per a day for 2 weeks	2 weeks	1) Effective rate 2) ROM 3) VAS	1) ER: $P<0.05$ 2) ROM: $P<0.05$ 3) VAS: $P<0.01$	Digestive symptoms such as nausea, loss of appetite, upper abdominal discomfort, and stomach pain in the comparison group
3	Su et al. (2014)	RCT	TMT (pushing technique on Ashi point, SJ-22, ST-7, GB-3, ST-6 for 5 minutes. Pinching technique on LI-4 for 1 minute.)	Ultrashort wave therapy	3 sessions per week for 2 weeks	2 weeks	ROM	ROM: $P>0.05$	NR
4	Su et al. (2013)	RCT	TMT (pushing and point-and-clic	Ultrashort wave therapy	3 sessions per week for 2 weeks	2 weeks	VAS	VAS: $P<0.05$	A small number of patients in the treatment group

			k technique on Ashi point, SJ−22, ST−7, GB−3, ST−6. Grasping technique on LI−4, SJ−5.)						experienced local swelling, skin redness and other symptoms. The symptoms disappeared after adjusting the intensity of the technique.
5	Gu et al. (2015)	RCT	TMT	semiconductor laser	Each treatment is about 20 minutes, every other day, 6 sessions as a course of treatment	Immediately after treatment	1) ER<br>2) Fricton's Craniomandibular Index<br>DI<br>PI<br>CMI<br>3) ROM	1) ER: P<0.05<br>2) Fricton's Craniomandibular Index<br>DI: P<0.05<br>PI: P<0.05<br>CMI: P<0.05<br>3) ROM: P<0.05	Not reported

CMI: Craniomandibular index, NR: Not reported, RCT: Randomized controlled trial, MD: Mean difference, RR: Relative risk, CMT: Chuna manual therapy, TMT: Tuina manual therapy, UC: Usual care,VAS: Visual Analogue Scale, NRS: Numeric Rating Scale, ROM: Range of Motion, BDI: Beck's Depression Inventory, JFLS: Jaw Functional Limitation Scale, PGIC: Patient Global Impression of Change, SF-12: Short Form-12 Health Survey, EQ-5D-5L: 5-Level EuroQol-5 Dimension, ER: Effective rate, FPSC: Facial Pain Score Scale, DI: Dysfunction index, PI: palpation index, TDP: Te Ding Dian Zi Bo Pu,

Eq. (E.8) [Q7] The Concurrent Treatment Group of Chuna Manipulation and usual Conservative Treat−ment VS the usual Conservative Treatment Group

No.	Study ID	Study design	Intervention (n)	Comparison (n)	Duration	F/U	Outcome measurements	Results	Adverse events
1	Gu et al. (2015)	RCT	TMT + TDP	TDP	daily for 10 days, 5 days interval, daily for 10 days	Immediately after treatment	1) ER	1) ER: P<0.05	NR
2	Li et al. (2008)	RCT	TMT + Very High Frequency	Very High Frequency	1 session per a day for 10 days	Immediately after treatment	1) ER	1) ER: P<0.05	NR

CMI: Craniomandibular index, NR: Not reported, RCT: Randomized controlled trial, MD: Mean difference, RR: Relative risk, CMT: Chuna manual therapy, TMT: Tuina manual therapy, UC: Usual care,VAS: Visual Analogue Scale, NRS: Numeric Rating Scale, ROM: Range of Motion, BDI: Beck's Depression Inventory, JFLS: Jaw Functional Limitation Scale, PGIC: Patient Global Impression of Change, SF-12: Short Form-12 Health Survey, EQ-5D-5L: 5-Level EuroQol-5 Dimension, ER: Effective rate, FPSC: Facial Pain Score Scale, DI: Dysfunction index, PI: palpation index, TDP: Te Ding Dian Zi Bo Pu,

Eq. (E.9) [Q8] Concurrent Treatment Group of Chuna Manipulation and Korean Medicine Treatment VS Acupuncture or Herbal Medicine Treatment Group

No.	Study ID	Study design	Intervention (n)	Comparison (n)	Duration	F/U	Outcome measurements	Results	Adverse events
1	Ding et al. (2015)	RCT	TMT + Acupuncture with moxibustion	Acupuncture with moxibustion	20 sessions for 20 days	Immediately after treatment	1) ER<br>2) VAS	1) ER: P<0.05<br>2) VAS: P<0.05	Not reported
2	Liu et al. (2013)	RCT	TMT + Herbal medicine	1) Herbal medicine<br>2) TMT	1) TID for 2 weeks<br>2) 1 session per a day for 2 weeks	1 week and 2 weeks	1) ER	1) ER<br>1 week: Not reported<br>2 weeks: Not reported	Not reported
3	Wan et al. (2014)	RCT	TMT + Acupuncture	1) Acupuncture<br>2) injection on acupoint	1 session per a day for 10 days	Immediately after treatment	1) ER	1) ER: P<0.05	Not reported
4	Bu et al. (2011)	RCT	TMT + electroacupuncture	electroacupuncture	1 session a day, 10 sessions as a course of treatment, 2 days apart, 4 courses in total	Immediately after treatment	1) ER<br>2) effectiveness for myofacial pain<br>3) effectiveness for external pterygoid muscle spasm	1) ER: P<0.05<br>2) effectiveness for myofacial pain: P<0.05<br>3) effectiveness for external pterygoid muscle spasm: P<0.05	Not reported
5	Jin et al. (2011)	RCT	CMT + Acupuncture Pharmacopuncture	Acupuncture Pharmacopuncture	2 sessions a week for 4 weeks	Immediately after treatment	1) VAS<br>2) FPSC	1) VAS: P<0.05<br>2) FPSC: P<0.05	Not reported

CMI: Craniomandibular index, NR: Not reported, RCT: Randomized controlled trial, MD: Mean difference, RR: Relative risk, CMT: Chuna manual therapy, TMT: Tuina manual therapy, UC: Usual care,VAS: Visual Analogue Scale, NRS: Numeric Rating Scale, ROM: Range of Motion, BDI: Beck's Depression Inventory, JFLS: Jaw Functional Limitation Scale, PGIC: Patient Global Impression of Change, SF-12: Short Form-12 Health Survey,

EQ-5D-5L: 5-Level EuroQol-5 Dimension, ER: Effective rate, FPSC: Facial Pain Score Scale, DI: Dysfunction index, PI: palpation index, TDP: Te Ding Dian Zi Bo Pu,

Eq. (E.10) [Q9] Herbal Medicine Treatment Group VS usual Conservative Treatment Group

No.	Study ID	Study design	Intervention (n)	Comparison (n)	Duration	F/U	Outcome measurements	Results	Adverse events
1	Hu et al. (2017)	RCT	Herbal medicine	medicine (Mesulide dispersible tablets BID, Sodium hyaluronate injection Q.WK)	2 weeks for a course, 6 course for treatment	12 weeks	1) ER	1) ER: P<0.05	Not reported

CMI: Craniomandibular index, NR: Not reported, RCT: Randomized controlled trial, MD: Mean difference, RR: Relative risk, CMT: Chuna manual therapy, TMT: Tuina manual therapy, UC: Usual care,VAS: Visual Analogue Scale, NRS: Numeric Rating Scale, ROM: Range of Motion, BDI: Beck's Depression Inventory, JFLS: Jaw Functional Limitation Scale, PGIC: Patient Global Impression of Change, SF-12: Short Form-12 Health Survey, EQ-5D-5L: 5-Level EuroQol-5 Dimension, ER: Effective rate, FPSC: Facial Pain Score Scale, DI: Dysfunction index, PI: palpation index, TDP: Te Ding Dian Zi Bo Pu

Eq. (E.11) [Q10] Concurrent Treatment Group of Herbal Medicine Treatment and usual Conservative Treatment VS usual Conservative Treatment

No.	Study ID	Study design	Intervention (n)	Comparison (n)	Duration	F/U	Outcome measurements	Results	Adverse events
1	Yan et al. (2015)	RCT	Herbal medicine TID + Very High Frequency QD (Western ultrashort wave treatment)	Very High Frequency QD (Western ultrashort wave treatment)	Herbal medicine for 7~14 days Very High Frequency QD for 10 days	Immediately after treatment	1) ER	1) ER: P<0.05	Not reported
2	Li et al. (2018)	RCT	Herbal medicine BID + local injection therapy	local injection therapy (Lidocaine hydrochloride 2 ml, triamcinolone acetonide 20 mg, vitamin b12 0.5~1mg and normal saline BI.WK)	4 weeks	4 weeks	1) ER<br>2) VAS<br>3) ROM<br>4) serum IL-6, IFN-γ, IL-1β	1) ER: P<0.05<br>2) VAS: P<0.05<br>3) ROM: P<0.05<br>4) serum IL-6, IFN-γ, IL-1β: P<0.05	Not reported

CMI: Craniomandibular index, NR: Not reported, RCT: Randomized controlled trial, MD: Mean difference, RR: Relative risk, CMT: Chuna manual therapy, TMT: Tuina manual therapy, UC: Usual care,VAS: Visual Analogue Scale, NRS: Numeric Rating Scale, ROM: Range of Motion, BDI: Beck's Depression Inventory, JFLS: Jaw Functional Limitation Scale, PGIC: Patient Global Impression of Change, SF-12: Short Form-12 Health Survey, EQ-5D-5L: 5-Level EuroQol-5 Dimension, ER: Effective rate, FPSC: Facial Pain Score Scale, DI: Dysfunction index, PI: palpation index, TDP: Te Ding Dian Zi Bo Pu,

Group

Eq. (E.12) [Q11] Concurrent Treatment Group of Herbal Medicine Treatment with Korean medicine Treat−ment VS Korean Medicine Treatment

No.	Study ID	Study design	Intervention (n)	Comparison (n)	Duration	F/U	Outcome measurements	Results	Adverse events
1	Yang et al. (2016)	RCT	Herbal medicine TID + Acupuncture	Acupuncture	Not reported	Not reported	1) ER<br>2) Scale score<br>① Subjective outcome<br>② Subjective outcome<br>3) VAS	1) ER: P<0.05<br>2) Scale score<br>①주관지표: 그룹간 비교 없음 (그룹내 치료 전후 비교만 있음)<br>②객관지표: 그룹간 비교 없음 (그룹내 치료 전후 비교만 있음)<br>3) VAS: P<0.05	Not reported
2	Hao et al. (2012)	RCT	Herbal medicine + Acupuncture, Laser QD	Acupuncture, Laser QD	20 days	20 days	1) ER	1) ER: No between-group comparison (Intervention: 85.71%, Comparison: 76.00%)	Not reported

CMI: Craniomandibular index, NR: Not reported, RCT: Randomized controlled trial, MD: Mean difference, RR: Relative risk, CMT: Chuna manual therapy, TMT: Tuina manual therapy, UC: Usual care,VAS: Visual Analogue Scale, NRS: Numeric Rating Scale, ROM: Range of Motion, BDI: Beck's Depression Inventory, JFLS: Jaw Functional Limitation Scale, PGIC: Patient Global Impression of Change, SF-12: Short Form-12 Health Survey, EQ-5D-5L: 5-Level EuroQol-5 Dimension, ER: Effective rate, FPSC: Facial Pain Score Scale, DI: Dysfunction index, PI: palpation index, TDP: Te Ding Dian Zi Bo Pu,

Eq. (E.13) [Q12] Exercise Treatment Group VS Inactive Control Group

No.	Study ID	Study design	Intervention (n)	Comparison (n)	Duration	F/U	Outcome measurements	Results	Adverse events
1	Yoda et al. (2003)	RCT	Exercise of TMJ	no treatment	3 months	After treatment	1) Success rate<br>2) Disk position	1) Intervention group: 61.9% improved. Comparison group: 0% improved (p=0.0001)<br>2) 23.1% improved	Not reported
2	Yoshida et al. (2011)	RCT	Exercise of TMJ	no treatment	NR	NR	1) Maximum mouth opening<br>2) Lateral movement compared with the opposite side<br>3) Lateral movement to affected side<br>4) protrusion<br>4) Success rate	There was a significant difference between the experimental (50/74, 68%) and control groups (3/74, 4%) in the degree of increased mouth-opening.	Not reported
3	Bae et al. (2013)	RCT	Exercise of TMJ (relaxation exercise)	no treatment	4 weeks	After treatment	1) ROM<br>2) Deviation<br>3) Occlusion<br>4) Pain	ROM, deviation and pain showed statistically significant in improvements after the intervention in the active exercise and relaxation exercise for the masticator muscle groups.	Not reported
4	Barbosa et al. (2019)	RCT	masticatory muscles focused	placebo (simulated laser therapy)	4 weeks	8 weeks	1) VAS<br>2) Efficiency<br>3) Time until	Pain scores decreased for both groups, but the	No adverse effects were reported

			endurance exercises				fatigue	intervention group showed lower values at 8 weeks.	
5	Lee et al. (2019)	RCT	Cervical Stabilization Exercise	placebo (simulated electrode pads)	4 weeks	6 weeks	1) Therabite range of motion scale 2) Maximum mouth oping	The upper cervical stabilization group showed more significant effect than the control group	Not reported

CMI: Craniomandibular index, NR: Not reported, RCT: Randomized controlled trial, MD: Mean difference, RR: Relative risk, CMT: Chuna manual therapy, TMT: Tuina manual therapy, UC: Usual care,VAS: Visual Analogue Scale, NRS: Numeric Rating Scale, ROM: Range of Motion, BDI: Beck's Depression Inventory, JFLS: Jaw Functional Limitation Scale, PGIC: Patient Global Impression of Change, SF-12: Short Form-12 Health Survey, EQ-5D-5L: 5-Level EuroQol-5 Dimension, ER: Effective rate, FPSC: Facial Pain Score Scale, DI: Dysfunction index, PI: palpation index, TDP: Te Ding Dian Zi Bo Pu

Eq. (E.14) [Q15] LLLT VS Inactive Control Group

Study	Country (period)	Interventions	Comparison	Disease of participants (Age : Mean±S.D)	Number of Participants	Treatment area	Session or Treatment period	Relevant outcomes
Amanat et al.(2013)	Iran	LLLT(980nm, 3000Hz and 12.73J/cm^2	sham laser	orofacial pain	IG: 30<br>CG: 30	Trigger point<br>Foci along the line of pain<br>(In the absence of a trigger point)	10 sessions (3 sessions per week)	VAS
Demirkol et al.(2015)	Turkey	LLLT(1064nm, 8 j/cm2, 0.25W, 20s)	Occlusal splint/ placebo	TMD characterized with myofacial pain	IG: 10<br>CG: 20<br>(or 10:10:10)	Muscle trigger point	5 times per week, for a total of 10 sessions	VAS
Cavalcanti et al.(2016)	Brazil	LLLT(780nm, 35.0 J/cm2,70mW, 20s)	PDP therapy/ Placebo therapy	Pain associated with TMD	IG: 20<br>CG: 40<br>(or 20:20:20)	(intraoral) pterygoid medial, (extraoral) intra-headset, pre-headset, gonion, anterior superior insertion of masseter	every other day, except weekends, for four weeks	P(Presence) or A(Absence) of pain
Costa et al.(2017)	Brazil	PBMT(Phtobiomodulation therapy)(830 nm, 100mW, 100 J/cm2 28s)	placebo	myalgia treatment of masticatory muscles	IG: 30<br>CG: 30	bilaterally to specific points on the masseter and temporal muscles	in a single day	VAS
Decarli et al.(2012)	Brazil	LLLT(830nm, 100nW, 100 Jcm2, 28s) and piroxicam	active laser and placebo piroxicam/ placebo laser and piroxicam	temporomandibular joint arthralgia	IG: 11<br>CG: 21<br>(or 11:11:10)	10 temporomandibular joint and muscle points on each side (joint capsule (lateral, posterior, superior, anterior, inferior), masseter (origin,	four sessions (each session is twice a week, over a	VAS

Study	Country (period)	Interventions	Comparison	Disease of participants (Age : Mean±S.D)	Number of Participants	Treatment area	Session or Treatment period	Relevant outcomes
						insertion) and temporal (anterior, middle, posterior).)	10-day period,	
Devecchio et al. (2019)	Italy	LLLT(808nm, 5 J/min, 250mW)	sham laser	mono or bilateral TMJD	IG: 30<br>CG: 60<br>(or Study Group: Placebo Group: Drug Group = 30:30:30)	pain area	1 week, twice daily	VAS
Fornaini et al. (2015)	Italy	LLLT(808nm, 250mW, 35 J/cm2, 15 min)	inactive laser	mono- or bi-lateral TMD	IG: 12<br>CG: 12	irradiation of the cutaneous zone corresponding to the TMJ each side	once a day for 2 weeks	VAS
Herpich et al.(2017)	Brazil	phototherapy with a combination of super-pulsed laser (905 nm), red (640 nm), and infrared (875 nm) light emitting diodes	Placebo	asseter and temporal muscles in women with temporomandibular disorder(TMD).	Group 1 - 2.62 J;<br>Group 2 - 5.24 J;<br>Group 3 - 7.86 J; placebo group.<br>(15: 15: 15: 15)	masseter (three points) and temporal (two points) muscles bilaterally	(한번 쏘고 24시간 마다 사후관찰 한 듯)	VAS, EMG
Leadegodoy et al.(2017)	Brazil	LLLT(780nm, 50mW, 25 J/cm2, 20s)	Sham laser	with chronic temporomandibular disorders (TMDs)	IG: 9<br>CG: 7	masseter & anterior temporal muscle	12 sessions	RMS(Root Mean Square), EMG
Machado et al.(2016)	Brazil	LLLT(780nm, 60mW, 60± 1.0 J/cm²,	No intervention	with chronic temporomandibular disorders(TMDs)	GI: 21<br>GII: 21<br>GIII: 19	five sites in the TMJ region: lateral pole; superior, anterior, posterior, and inferior	The treatment sessions lasted for 45	Orofacial Myofunctional Evaluation with Scores

Study	Country (period)	Interventions	Comparison	Disease of participants (Age : Mean±S.D)	Number of Participants	Treatment area	Session or Treatment period	Relevant outcomes
		40s)groups: GI (LLLT + O M exercises), GII (orofacial myofunctional therapyOMT GIII (LLLT placebo +OMexercises) GIV (LLLT)			GIV: 18 GC; 20	points of the condylar position; and on the painful sites of the masseter and temporal muscles reported by the subjects	min and were held on a weekly basis during the first 60 days and on a biweekly basis thereafter for a total of 12 sessions, totaling a maximum of 9 h in the 120-day period.	(OMES) Protocol
Magri et al.(2019)	Brazil	LLLT(780nm) ·masseter and anterior temporal = 5 J/cm2 (20 mW - 10 s) ·TMJ area = 7.5 J/cm2 (30 mW - 10 s)	placebo	with painful TMD (31.7 ±5.2 years)	IG: 20 CG: 21	the masseter (three points: upper, middle, and lower), the anterior temporalis (three points: upper, middle, and lower), and the temporomandibular joint (TMJ) region (four points forming a cross and one central point)	two sessions per week for four consecutive weeks, totaling eight sessions.	VAS
Magri et al.(2017)	Brazil	LLLT(780 nm; masseter and anterior temporal = 5 J/cm2, 20 mW, 10 s; TMJ area = 7.5 J/cm2,	placebo	with temporomandibular disorders (TMD) frequently	laser: 31 placebo: 30 controls: 30	the masseter (three points: upper, middle, and lower), the anterior temporal (three points: upper, middle, and lower), and the TMJ region (four points forming a cross and one central point)	twice a week, eight sessions	VAS

Study	Country (period)	Interventions	Comparison	Disease of participants (Age : Mean±S.D)	Number of Participants	Treatment area	Session or Treatment period	Relevant outcomes
		30 mW, 10 s						
Nadershah et al.(2019)	India	Photobiomodulation therapy(940nm, 7W, 300J, 2min; 24s per application point)	sham laser	with unilateral TMJ and masticatory muscles pain during function	IG: 108<br>CG: 94	2 cm distance from the skin to 5 points at the temporal (center of Temporalis muscle), zygomatic (origin of Masseter muscle), angle of the mandible (insertion of Masseter muscle), pre-auricular, and mastoid areas.	every 48 h for 10 days	VAS
Abreuvenacio et al.(2005)	Brazil	LILT(low intensity laser therapy) (780nm, 30mW, 10s 6.3 J/cm2)	placebo	presenting temporomandibular joint (TMJ) pain and mandibular dysfunction	IG:15<br>CG: 15	three points in each TMJ	six sessions	VAS
Venezian et al.(2005)	Brazil	Diode laser(780nm) Group I-dose of 25 J/cm2 (50mW for 20 seconds, *actual treatment); Group II-dose of 25 J/cm2* (50mW for 20 seconds, *placebotreat*	placebo	with myofascial pain (41.58)	The four groups had 12 components each (12:12:12:12)	temporalis and masseter muscles	twice a week (four weeks	VAS

Study	Country (period)	Interventions	Comparison	Disease of participants (Age : Mean±S.D)	Number of Participants	Treatment area	Session or Treatment period	Relevant outcomes
		ment); Group III-dose of 60 J/cm2 (60mW for 40 seconds, *actual treatment) and GroupIV-dose of 60 J/cm2* (60mW for 40 seconds *placebo treatment)*						
Yamaner et al.(2020)	Turkey	LLLT(820 nm, 3 J/cm2, 300 mW, 10s)	sham laser	TMD with disc displacement with reduction(DDR) 31.51 ±10.32	IG: 33 CG: 29 randomized in a 1:1 ratio into one of two groups: (1) treatment or (2) placebo. Then, 40 TMJs in the reatment group were randomized in a 1:1 ratio into one of two subgroups: (1)laser or (2) ozone, and 40 TMJs in the placebo group were randomized in a 1:1 ratio into	predetermined TMJ points(anterior aspect, posterior aspect, and joint interface)	three times per week for 10 min, for a total of six sessions	VAS

Study	Country (period)	Interventions	Comparison	Disease of participants (Age : Mean±S.D)	Number of Participants	Treatment area	Session or Treatment period	Relevant outcomes
					one of two subgroups: (1) sham laser or (2) sham ozone.			
Ahrai et al.(2014)	Iran	LLLT(average power 50 mW, peak power 80 W, 1,500 Hz, 120 s, 6 J, and 3.4 J/cm2 per point)	placebo	20 female patients with myogenic TMD(mean age of 35.5 years.)	IG: 10<br>CG: 10	masseter muscle; anterior, middle, and posterior portions of the body of the temporalis muscle; and insertion of the internal pterygoid muscle	three times a week for 4 weeks	VAS
Cetiner et al.(2006)	Turkey	Class IIIb laser product (wavelength, 830 nm; duration, 162 sec; dosage, 7 J/cm2	placebo	myogenic originated TMD(Age : 31.7 years)	IG : 24<br>CG : 15	joint capsule (lateral, posterior, superior), masseter (anterior, inferior, deep), temporal (anterior, deep, middle, origin), medial, and lateral pyterigoid muscles bilaterally	10 sessions daily for 2 weeks, excluding weekends	VAS, Number of tender points, Maximum mouth opening, lateral motion
da Cunha et al.(2008)	Brazil	LLLT(830nm, 500mW, 20s, 4J/point))	placebo	who presented for diagnosis and treatment of TMD	IG : 20<br>CG : 20	painful area	once a week for four consecutive weeks	VAS, Craniomandibular Index
da Silva et al.(2012)	Brazil	G-I: 780 nm, 70 mW, IA-TMD submitted	placebo	presenting signs and symptoms associated with TMD for over six months	IG : 30<br>CG : 15<br>(15 : 15 : 15)	anterior, superior, posterior, posteroinferior points of the condylar	two weekly sessions during five weeks,	VAS

Study	Country (period)	Interventions	Comparison	Disease of participants (Age : Mean±S.D)	Number of Participants	Treatment area	Session or Treatment period	Relevant outcomes
		to an energy dose of 52.5 J/cm2; G-II: 780 nm, 70 mW, dose of 105.0 J/cm2		(Age : 39.7 years)		position, and in the external auditory meatus, three points on the masseter muscles, and one point on the anterior temporalis muscles	totaling 10 applications	
emshoff et al.(2008)	Austria	LLLT (Helium Neon, 632.8 nm, 30 mW, 1.5 J/cm2)	sham LLLT	unilateral TMJ pain (Age : 42.9 years)	IG : 26<br>CG : 26	the skin at the center of the upper joint space, approximately 1 cm in front of the tragus	2 to 3 treatments per week for 8 weeks	VAS
kulekciogl u et al.(2003)	Turkey	LLLT(904nm, 17mW, 1000 Hz, duration: 180 seconds, dosage: 3 J/cm2)	placebo	arthrogenic and myogenic TMD (Age : 37.0±12.3 years)	IG : 20<br>CG : 15	four most tender points selected during examination	fifteen sessions	VAS, TMJ MRI, Number of tender points, Number of joint sounds, Active/Passiv e mouth opening, lateral motion
Lasemi et al.(2008)	Iran	LLLT (980 nm, 80 Hz, 6 J) at three points over the TMJ (ie, 2 J	placebo	TMD	IG : 24<br>CG : 24	(1) the posterior aspect of the joint with the mouth open to treat the posterior articular branches of the	2 treatment sessions with a 48-h interval	VAS

Study	Country (period)	Interventions	Comparison	Disease of participants (Age : Mean±S.D)	Number of Participants	Treatment area	Session or Treatment period	Relevant outcomes
		per point and 1.5 J at the other sites of muscle pain)				auriculotemporal nerve (2) an area anterior to condyle in the sigmoid notch with the mouth closed for the area of insertion of the lateral pterygoid muscle into the condylar neck and meniscus (3) the joint interface with mouth open		
Marini et al.(2010)	Italy	SLLLT(frequency range 1 to 50 kHz, wave length 910 nm, mean power 400mW, and peak power 45W. 1. 20 kHz for 10 minutes 2. 18 kHz for 5 minutes 3. 16 kHz for 5 minutes)	ibuprofen / placebo	TMJ DD without reduction and osteoarthritis, pain for more than 6 months of similar intensity	IG : 39<br>CG : 60<br>(39 : 30 : 30)	TMJ areas (정확하게 안적혀있음)	10 consecutive days (5 d/wk)	VAS

Study	Country (period)	Interventions	Comparison	Disease of participants (Age : Mean±S.D)	Number of Participants	Treatment area	Session or Treatment period	Relevant outcomes
Sancakli et al.(2015)	Turkey	LLLT (820nm, 300mW, 3J/cm2)	placebo	diagnosis of myofascial pain according to the Research Diagnostic Criteria for Temporomandibular Disorder (RDC/TMD)(Age : 39.2)	IG : 20<br>CG : 10<br>(10 : 10 : 10)	Group I : precisely and continuously to the greatest points of pain in the related muscle (masseter and/ or temporalis)<br><br>Group II : three predetermined points on the masseter muscle (superior [MS], middle [MM], and inferior [MI] points) and three points on the temporalis muscle (anterior [TA], middle, and posterior points).	three times per week, for a total of 12 sessions	VAS, muscle palpations, PPTs(kg/cm2) on the masticatory muscles, Mandibular movements
sattayut et al,(2012)	United kingdom	LILT(Group I : 820nm, 21.4J/cm^2, 4J/point, 60mW / Group II : 820nm, 107J/cm^2, 20J/point, 300mW)	placebo	unilateral myogenous TMD(Age : 35)	IG : 20<br>CG : 10<br>(10 : 10 : 10)	auriculotemporal nerve at the posterior aspect of the TMJ, trigger point on the masseter muscle	three times per week	pain pressure threshold
shirani et al.(2008)	Iran	LLLT(probe I : 660 nm, 17.3 mW, 6.2J/cm^2, 0Hz / probe II : 890nm, 9.8W,1J/cm^2, 1500Hz)	placebo	diagnosis of MPDS(age : 23.8 years)	IG : 12<br>CG : 4<br>(4 : 4 : 4 : 4)	medial and lateral pterygoid muscles	twice a week for 3 weeks	VAS

Study	Country (period)	Interventions	Comparison	Disease of participants (Age : Mean±S.D)	Number of Participants	Treatment area	Session or Treatment period	Relevant outcomes
		Group I : probe I + II Group II : probe I Group III : prboe II						
Wang Xiaodong et al.(2011)	China	LLLT (50Hz, 300mW, 650nm/830nm)	placebo	TMD	IG : 21<br>CG : 21	측면, 후면, 상부 관절낭	once a day for 6 consecutive days	VAS
Carraso et al,(2009)	Brazil	LILT(Group I : 780nm, 50mW, 25 J/cm2 / Group II : 780nm, 60mW, 60 J/cm2 / Group III : 780nm, 70mW, 105 J/cm2)	placebo	MPS(myofascial pain syndrome) and having one active trigger point in the anterior masseter and anterior temporal muscles	IG : 30<br>CG : 30<br>(10 : 10 : 10 : 10 : 10 : 10)	trigger point	twice a week, for four weeks	VAS
Carrasco et al.(2008)	Brazil	LILT(780 nm, 70 mw, 60s, 105J/cm2)	placebo	TMD	IG : 7<br>CG : 7	five points of the temporomandibular joint (TMJ) area: lateral point (LP), superior point (SP), anterior point (AP), posterior point (PP), and posterior-inferior point (PIP) of the condylar position	twice per week, for a total of eight sessions.	VAS, colorimetric capsule method
Contl et al.(1997)	Brazil	LLLT(100 rn W, 4J)	placebo	TMD	IG : 10<br>CG : 10	For the arthrogenous group, the probe was	once a week for three	VAS

Study	Country (period)	Interventions	Comparison	Disease of participants (Age : Mean±S.D)	Number of Participants	Treatment area	Session or Treatment period	Relevant outcomes
					(5 : 5 : 5 : 5)	placed over the lateral joint surface close at a degree angle to allow optimum joint penetration. For the myogenous group, the probe was applied over the most painful muscle spot, detected during muscle palpation	consecutive weeks	
Fikackova et al.(2007)	Czech	LLLT(Group I : 400mW, 830nm, 10 J/cm2 / Group II : 400mW, 830nm, 15 J/cm2)	sham laser(0.1 J/cm2)	TMD (Age : 41 years)	IG : 61 CG : 19 (33 : 28 : 19)	In front of the tragus, the meatus acusticus externus(when the mouth was open), 2 cm in front of the tragus, under the zygomatic arch(when the mouth was closed)	10 sessions within 1 month	two possible categories (successful and unsuccessful) for use in statistical analysis
de Moraes Maia et al.(2014)	Brazil	LLLT(808nm, 100mW, 70 J/cm2)	placebo	myofascial pain	IG : 12 CG : 9	trigger points of the anterior temporal and masseter muscles	two times per week for 4 weeks	masticatory performance, pressure pain threshold, VAS
madani et al.(2014)	Iran	LLLT(50mW, 1500Hz, 3.4 J/cm2)	placebo	TMJ osteoarthritis	IG : 10 CG : 10	posterior, anterior, and superior of the mandibular condyles, and inside the external auditory duct) origin, body, and insertion of the masseter muscle; anterior, middle, and posterior	three times a week for 4 weeks, totaling 12 sessions	VAS

Study	Country (period)	Interventions	Comparison	Disease of participants (Age : Mean±S.D)	Number of Participants	Treatment area	Session or Treatment period	Relevant outcomes
						parts of the body of the temporalis muscle; and insertion of the internal pterygoid muscle		
mazzetto et al.(2007)	Brazil	LILT(780 nm, 70 mW, 89.7 J/cm2)	placebo	TMD	IG : 24<br>CG : 24	external auditive duct toward the retrodiskal region, on both sides	twice a week for four weeks	VAS
Mazzetto et al.(2010)	Brazil	LLLT(830 nm, 40 mW, 5J/cm2)	placebo	TMD	IG : 20<br>CG : 20	condyle lateral pole: superior, anterior, posterior, and posterior-inferior	twice a week during 4 weeks	VAS
Frare et al.(2008)	Brazil	LLLT(904 nm, 15mW, 6 J/cm2)	placebo	TMD	IG : 10<br>CG : 10	pre-auricular region and external auditory meatus	twice a week, for four weeks	VAS

Eq. (E.15) [Q15] TENS VS Inactive Control Group

Study	Country (period)	Interventions	Comparison	Disease of participants (Age : Mean±S.D)	Number of Participants	Treatment area	Session or Treatment period	Relevant outcomes
Ferreira et al.(2016)	Brazil	TENS	Sham TENS	TMD (IG: 25.10±3.87 CG: 24.15±3.01)	IG: 15 CG: 15	Masseter muscles and anterior temporalis muscle	1 time	VAS, pain threshold
Seifi et al. (2017)	Iran	TENS	Sham TENS	TMD	IG: 10 CG: 10	Below the earhole, back of the neck	4 times	VAS, Maximum Mouth-Opening, Tenderness of Masticatory Muscle

Eq. (E.16) [Q16] TENS VS Conservative Therapy

Study	Country (period)	Interventions	Comparison	Disease of participants (Age : Mean±S.D)	Number of Participants	Treatment area	Session or Treatment period	Relevant outcomes
Kruger et al. (1998)	South Africa	TENS+conservative therapy (ibuprofen, biteplate, etc)	Conservative therapy	Myofacial pain dysfunction (38.5±14.65)	IG: 5<br>CG: 5	Trigger point areas in the masseter and temporalis muscles	14 weeks	Pain score
Shanavas et al. (2014)	India	TENS with medication	Medication	TMD (only range reported)	IG: 20<br>CG: 20	NR	5 days	VAS

Eq. (E.17) [Q17] Concurrent Treatment Group of Korean Physical Therapy and usual Conservative Treat-ment VS the usual Conservative Treatment Group

No.	Study ID	Study design	Country (period)	Intervention (n)	Comparison (n)	Disease of participants (Age : Mean±S.D)	Number of Participants	Session or Treatment period	Relevant outcomes	Results	Adverse events
1	Ritenbaugh et al. (2008)	RCT	USA (2001-2003)	TCM	Usual care	Temporomandibular disorders (IG: 40.1±8.5 CG: 40.5±9.4)	IG: 50 CG: 60	2 times with a week for 6 weeks	1) Pain 2) Impact on social life	TCM provided significantly greater decreases in average pain than SC.	No serious events
